# Characterisation of Antisense Oligonucleotides by Ion‐Pair Reversed‐Phase UHPLC‐HRMS: Method development using Design of Experiments

**DOI:** 10.1002/jms.70049

**Published:** 2026-03-19

**Authors:** Antonio Triolo, Fabiana Tavani, Prisca Barnini, Sandra Furlanetto, Serena Orlandini

**Affiliations:** ^1^ Laboratory of Advanced Analytics Menarini Ricerche Spa Florence Italy; ^2^ Department of Chemistry “Ugo Schiff” University of Florence Florence Italy

**Keywords:** antisense oligonucleotides, design of experiments, impurities characterisation, ion‐pair reversed‐phase UHPLC‐HRMS, response surface methodology

## Abstract

The quality control of therapeutic antisense oligonucleotides (ASOs) poses significant analytical challenges due to the complexity of their synthesis and degradation processes and the need to ensure the safety and efficacy of active pharmaceutical ingredients (APIs). In this study, an ion‐pair reversed‐phase ultra‐high‐performance liquid chromatography–high‐resolution mass spectrometry (IP‐RP‐UHPLC‐HRMS) method based on Orbitrap technology was developed using fomivirsen (FMV) and tofersen (TFR) as model compounds. A preliminary scouting phase was dedicated to selecting the type of ion‐pair reagent and MS parameters settings (sheath gas, auxiliary gas temperature and S‐lens), based on MS spectrum quality (charge state distribution, presence of adducts and in‐source fragments), MS signal height and chromatographic peak shape. Design of Experiments (DoE) through response surface methodology was employed to evaluate the effects of the following factors in depth: concentration of the ion pair reagent *N*,*N*‐diisopropylethylamine and of 1,1,1,3,3,3‐hexafluoroisopropanol in the mobile phase and elution gradient slope. The responses selected were the UV height and baseline width of the main peak of the APIs, as well as the resolutions between selected impurities from their extracted MS chromatograms. A multidimensional space (sweet spot) was defined in which the response requirements were met, enabling multicriteria optimisation and the set‐up of two distinct IP‐RP‐UHPLC‐MS methods for FMV and TFR characterisation, based on shared mobile phase components and MS parameters. All four FMV impurities and 13 out of 15 detected TFR impurities were identified above 0.1%. DoE approach has been demonstrated to be an effective tool for achieving a balance between sensitivity and selectivity in the analysis of ASOs, identifying optimum conditions tailored to oligonucleotide type and ion‐pair agent and paving the way for more structured development processes, as recommended by recent regulatory requirements for pharmaceutical analytical procedure development.

## 1 Introduction

Since the approval of fomivirsen (FMV) in 1998, antisense oligonucleotides (ASOs) have found growing use in the therapy of genetic, oncological, inflammatory and viral diseases [1]. ASOs are synthetically modified single‐stranded nucleic acid sequences, typically ranging from 18 to 30 nucleotides in length, with a molecular mass between 6 and 18 kDa [2]. They selectively bind to specific complementary mRNA targets, inhibiting the expression of genes involved in the targeted disease. ASOs are obtained by solid‐phase synthesis (SPS) and have undergone various modifications to their phosphodiester backbone, ribose moiety and nucleobases to enhance stability, resistance to nucleases, pharmacokinetic properties and binding affinity [3]. From a regulatory perspective, ASOs cannot be categorised as biotherapeutics or small‐molecule drugs due to their molecular mass, structural similarity to biological counterparts and synthetic origin. Consequently, applying current guidelines on manufacturing processes, characterisation, specifications and analytical control is not always feasible or appropriate [4]. Following the publication of position papers, the European Medicines Agency (EMA) has recently issued a draft guideline [5].

The quality control (QC) of the oligonucleotide (ON) full‐length product (FLP) employed as an active pharmaceutical ingredient (API) is more challenging than the QC of small‐molecule APIs due to the complexity of its synthesis and degradation processes, which leads to greater quantities of structurally similar impurities [6]. In particular, the monitoring of the main drug and of product‐related impurities is strictly connected not only to quality but even more to safety and efficacy for the patients and necessitates suitable analytical procedures [7]. Even if synthetic yields of 98%–99% are achieved at each synthesis step, the total amount of impurities tends to increase with the length of the ON chain, resulting in a highly complex impurity profile. Three distinct impurity classes can be defined: shortmers (deletion sequence *n* − 1, *n* − 2, etc.), longmers (addition sequences *n* + 1, *n* + x) and full‐length modified impurities. The first two classes differ in terms of charge, whereas the third differs in chemical structure due to various chemical modifications employed to stabilise the backbone, such as deamination and depurination. Additionally, variations may be produced by thermal stress, sulfur loss, partial oxidation during SPS, adduct formation as chloral and acrylonitrile adducts or other reactions [6, 8].

Overall, impurity characterisation of ONs poses significant analytical challenges for a variety of reasons. Firstly, impurities often occur in mixtures that are closely related to each other and to the FLP. For example, the *n* − 1 impurity is frequently not a single impurity, but rather a heterogeneous population in which any of the residues may be absent [7]. These compounds exhibit analogous physico‐chemical properties, molecular masses and similar UV absorption maxima (around 260 nm) [9]. Furthermore, the presence of an acidic phosphate (PO) or phosphorothioate (PS) group for each residue results in a net negative charge, rendering them highly polar and difficult to separate chromatographically. Another issue with PS oligonucleotides is the presence of diastereoisomers, which results in chromatographic peak broadening [4, 7].

For ASO characterisation, the use of orthogonal separation and/or detection methods is generally required. The most frequently employed method is HPLC‐UV/MS, predominantly conducted via electrospray ionisation in negative ion mode (ESI(−)) to take advantage of the deprotonation of the highly acidic phosphate groups [10]. More recently, the association of MS to ion mobility spectrometry has provided an additional orthogonal separation tool, compatible with chromatographic time scale. This enhances the overall analytical resolution; however, its use is still far from routine [11, 12]. Several analytical separation techniques for ONs have been used, such as ion‐exchange chromatography [13], capillary gel electrophoresis [14] and more recently mixed phase mode [15, 16] and hydrophilic interaction chromatography [17, 18]. Anyway, ion‐pair reversed‐phase HPLC (IP‐RP‐HPLC) is the most popular, due to its robustness, resolution capability and compatibility with MS detection [6, 8, 19, 20], although suffering from signal suppression and adduct ions formation due to the ion‐pair reagent (IPR) and other mobile phase components [21]. An extensive review on the characterisation of ON impurities and degradants by MS has been presented by Pourshahian [7].

Combining UV and MS detection takes advantage of their complementarity. ONs are conveniently detected by UV to determine the assay of the drug substance and quantify the impurities that are chromatographically separated from the FLP peak, with the advantage that ONs and related substances have similar weight‐based relative response factors [7]. However, UV does not provide information on impurities coeluting with the FLP, and peak identities are uniquely based on their retention times. Conversely, MS is the technique of choice for directly obtaining structural information from ONs and their impurities, even when complete separation of impurities from the FLP and from each other is not technically feasible, as it often occurs with several related substances such as the *n* − 1 deletion and *n* + 1 addition, mono‐phosphodiester variant (mono‐PO), *n* + protecting groups. In addition, MS detection is highly sensitive (less than 100 pg/mL), though MS quantitation may be affected by differences in relative response factors [22].

High‐resolution MS (HRMS) instruments are usually necessary due to the appearance of ON ESI spectra as a series of multiply charged ions with isotopic peaks that differ by only 0.1–0.2 Da. In fact, lack of chromatographic separation may result in signal overlap between unseparated impurities of similar mass, which can be much more confidently resolved than with low‐resolution instruments. Furthermore, the low‐ppm mass accuracy of HRMS provides reliable information on the identity and integrity of analytes, as well as the presence of chemical modifications. Detailed information on their sequence and the localisation of site‐specific modifications can be obtained through tandem MS of the unfragmented ONs [23, 24, 25].

IP‐RP‐HPLC coupled to ESI‐MS using mobile phases containing alkylamine and an acidic modifier has emerged as the prevailing method for analysing product‐related impurities in ON drug products; however, the originally used formic and acetic acids generated ion suppression phenomena [6, 19, 26]. Apffel et al. introduced a different acidic modifier, HFIP, buffered with TEA. The presence of the strong organic base TEA suppresses sodium and potassium adducts to the phosphate backbone of nucleic acids, and the optimised HFIP/TEA system enhances electrospray ionisation efficiency, leading to improved signal‐to‐noise ratios, sharper peaks and better chromatographic resolution and MS sensitivity [27]. Since then, several systematic studies compared the effect of different alkylamines as IPRs on signal intensity [28, 29, 30, 31]. Alternative fluorinated alcohols were also evaluated to optimise signal intensity, structural preservation and compatibility with different IPRs. It was highlighted that the best solvent system must be tailored to both the ON type and the IPR, because the physico‐chemical properties of IPRs and the characteristics of ONs jointly influence the ESI desorption process, thereby affecting LC‐ESI‐MS sensitivity [31, 32]. Nowadays, the most common mobile phase components employed to enhance MS sensitivity are secondary or tertiary alkylamines as IPRs, combined with fluoroalcohols, at a defined concentration and ratio [6]. However, the intricate interplay between the mobile phase components, LC‐MS instrumental parameters and the sequence and composition of the ONs affects the separation, spectral quality and sensitivity of detection, often resulting in a challenging method set‐up and complicated data processing procedures [33]. Therefore, fine‐tuning of the experimental parameters is required to ensure efficacy, and a systematic development approach is recommended [6, 34].

In most cases, the IP separation of ONs has been achieved using C18 columns [6]. A study comparing HPLC and UHPLC columns of different lengths and chemistries in the stationary phase, with variously sized particles, revealed that long C18 columns with small particle sizes, held at elevated temperatures, provided the best resolution for phosphorothioate and phosphate ON impurities [35]. On the opposite, Fekete et al. suggested the use of very short columns (20 mm), based on the general ‘bind and elute’ retention behavior of model ss‐DNAs ranging from 10 to 100 base length, also pointing out the importance of avoiding adsorption phenomena on metallic surfaces inside the column and the entire flow path [34]. These different findings are not surprising, considering the different analyte classes that are the object of the studies, and point out once more the importance of developing methods tailored to the specific analyte under study.

**FIGURE 1:**
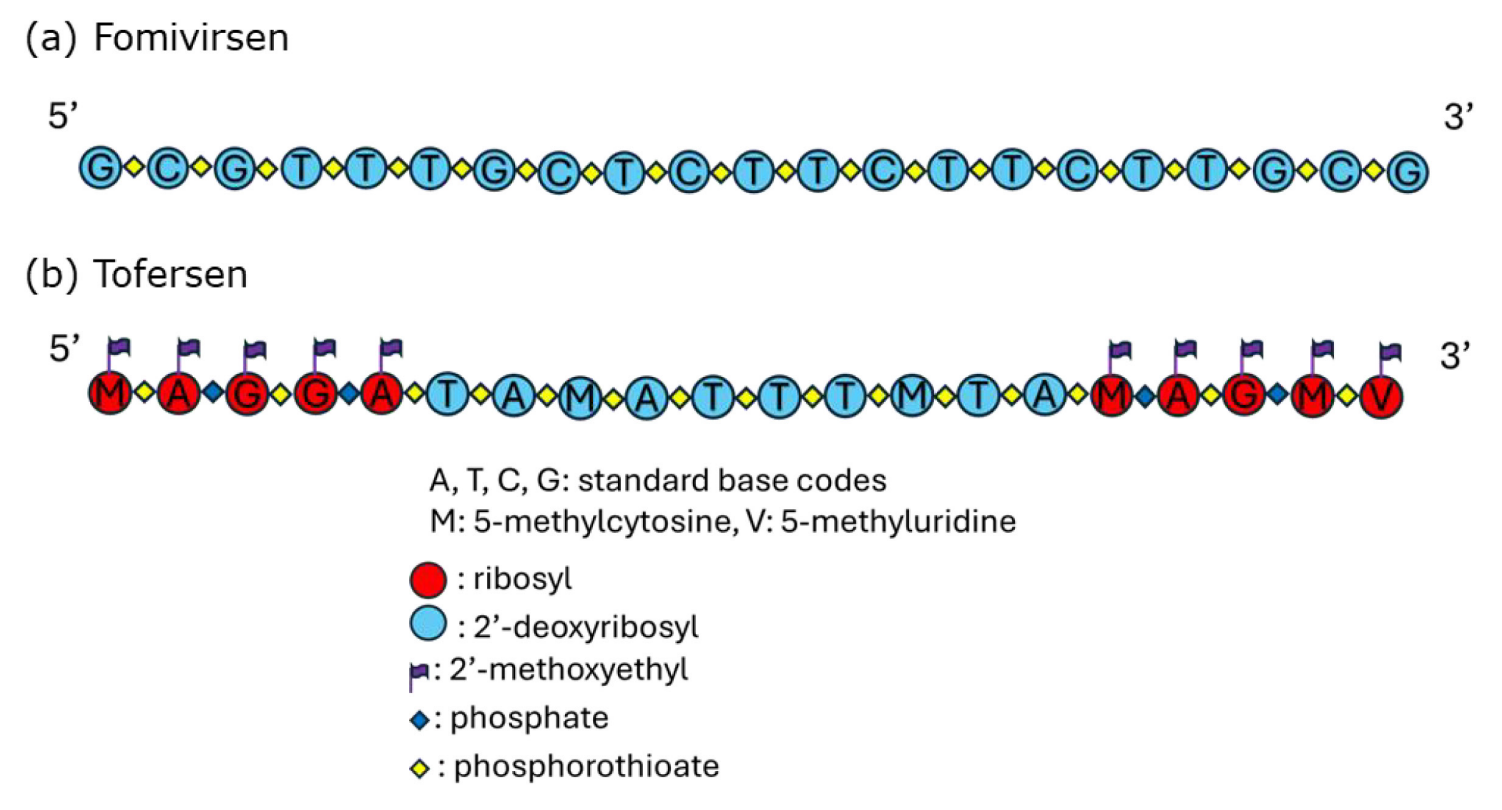
Graphical representations of the molecular structures of (a) fomivirsen and (b) tofersen.

In the effort of setting up a general and possibly simplified procedure for the analysis of ASOs and impurities, the aim of this work was to develop an IP‐RP‐UHPLC‐HRMS method using FMV and tofersen (TFR) as model compounds, through a Thermo Vanquish UHPLC chromatograph with diode‐array UV detector, interfaced to a Thermo Q Exactive Plus mass spectrometer, equipped with Orbitrap technology. The Orbitrap is particularly well suited for such analysis due to its high resolution and sensitivity, enabling the differentiation of molecules with very similar masses, which is crucial for the identification and quantification of ON impurities. FMV (Vitravene) was the first ASO approved by both the US Food and Drug Administration (FDA) and EMA for clinical use in 1998 for the treatment of Cytomegalovirus retinitis in HIV patients. It is a DNA‐based oligonucleotide, a 21‐mer ASO linked by PS‐modified backbone. TFR (Qalsody) received accelerated approval by the FDA in 2023 to treat patients with amyotrophic lateral sclerosis associated with a mutation in the superoxide dismutase 1 gene (SOD1‐ALS). It is a gapmer designed with 2′ modified nucleotides at both the 5′ and 3′ ends to enhance their nuclease resistance and affinity for target mRNAs, but the central portion is designed with deoxynucleotides to support RNase‐H1‐mediated cleavage [3]. The graphical representations of the structures of FMV and TFR are reported in Figure 1. To the best of our knowledge, no analytical procedure has been developed specifically to address the characterisation of these ASOs and their impurities.

Considering the task to develop an analytical procedure as general as possible, tailored to both the ON model compounds, and the complexity of the interactions of mobile phase components and LC‐MS instrumental parameters, unavoidably leading to the search of a compromise between chromatographic resolution and MS sensitivity [33], this manuscript focuses on the use of Design of Experiments (DoE) [36] as a key tool for developing the analytical procedure. DoE improves the reliability and robustness of analyses and meets the regulatory requirements of the International Council for Harmonisation (ICH) in the pharmaceutical field [37]. It is also a pillar of Quality by Design, an approach introduced by regulatory authorities to ensure the quality of pharmaceutical processes and products and recently recommended for the development of analytical procedures [37, 38, 39].

Analytical Quality by Design (AQbD) has been widely utilised to develop separation procedures for QC of small‐molecule APIs [40, 41]. However, to date, only one paper has focused on applying AQbD principles to the development of an LC‐HRMS procedure for the analysis of ASOs, specifically for the assay of nusinersen [9]. The present work intends to expand the application of DoE to provide a comprehensive overview of ASO quality, focusing on impurity profile and characterisation. This serves as an introduction to more structured AQbD‐compliant development procedures, as the field of ON analysis encourages the adoption of systematic approaches and requires more examples [6, 34].

In this study, a preliminary scouting phase was dedicated to the selection of the type of IPR and MS parameter settings, focusing the research on the MS spectrum quality (charge state distribution, presence of adducts and in‐source fragments), the height of the MS signal and the chromatographic peak shape. In a subsequent step, DoE through response surface methodology (RSM) was employed to evaluate in‐depth the effects of the chosen IPR and acidic modifier concentrations, and of the elution gradient slope, on the chromatographic resolution of selected impurities and the UV detection sensitivity. Through Box–Behnken design, a multidimensional space (sweet spot) was defined, where the responses were simultaneously optimised. Two distinct IP‐RP‐UHPLC‐MS methods were developed for FMV and TFR based on shared mobile phase components and MS parameters. The developed methods enabled the detection and relative quantitation of the occurring impurities down to about 0.1% level in both model ONs. In addition, all four FMV impurities and 13 out of 15 detected TFR impurities were identified.

## 2 Materials and Methods

### 2.1 Chemicals and Reagents

FMV (sodium salt, purity > 99%) and TFR (sodium salt, purity 95.5%) were obtained from MedChemExpress LLC (Monmouth Junction, NJ, USA). The samples were non‐commercial/non‐pharmaceutical grade and were expected to contain all possible impurities. FMV, whose structure is reported in Figure 1a, is a PS‐modified oligonucleotide consisting of 21 nucleotides with a specific sequence: 5′‐Gd‐sCd‐sGd‐sTd‐sTd‐sTd‐sGd‐sCd‐sTd‐sCd‐sTd‐sTd‐sCd‐sTd‐sTd‐sCd‐sTd‐sTd‐sGd‐sCd‐sGd‐3′. Its molecular formula is C_204_H_243_N_63_Na_20_O_114_P_20_S_20_, with a monoisotopic mass of 6677.5886 Da. The PS bonds instead of PO bonds make it more resistant to degradation by nucleases.

TFR, whose structure is shown in Figure 1b, is a 20‐residue oligonucleotide with a gapmer structure and a mixed RNA–DNA–RNA backbone (5–10–5). Its specific sequence is 5′‐Mw‐sAw‐pGw‐sGw‐pAw‐sTd‐sAd‐sMd‐sAd‐sTd‐sTd‐sTd‐sMd‐sTd‐sAd‐sMw‐pAw‐sGw‐pMw‐sVw‐3′; its molecular formula is C_230_H_317_O_123_N_72_P_19_S_15_, with a monoisotopic mass of 7123.1589 Da. TFR contains 19 inter‐nucleotide bonds, 15 of which are phosphorothioates and 4 of which are phosphates. Ten of the 20 residues in the central region contain 2′‐deoxy‐D‐ribose (central gap), whereas the remaining residues contain 2′‐O‐(2‐methoxyethyl)‐D‐ribose (2′‐MOE), arranged at the 5′ and 3′ ends and surrounding the central gap. Triethylamine (TEA), dibutylamine (DBA), *N*,*N*‐diisopropylethylamine (DIPEA), 1,1,1,3,3,3‐hexafluoroisopropanol (HFIP) and LC‐MS‐grade methanol (MeOH) were purchased from Sigma‐Aldrich (Milan, Italy). Ultrapure water was obtained with a MilliQ apparatus (Merck KGaA, Darmstadt, Germany).

### 2.2 Preparation of Standards and Sample

Standard stock solutions of TFR and FMV were prepared by accurately weighing (1 mg) and dissolving each compound in 1 mL of ultrapure water to make a 1 mg mL^−1^ solution. Twenty 50 μL aliquots were then taken, and the resulting samples were stored at −70°C. Prior to analysis, the samples were thawed, and 950 μL of water was added to achieve a final concentration of 50 μg mL^−1^. The thawed samples were freshly used.

### 2.3 Instrument and Equipment

Instrumental analysis was performed by a Vanquish Horizon UHPLC system (Thermo Fisher Scientific, Waltham, MA, USA), configured with a diode array detector, a binary pump, an autosampler and a thermostatted column compartment. The Vanquish system was coupled with a Q Exactive Plus BioPharma mass spectrometer equipped with a quadrupole‐Orbitrap analyser (Thermo Fisher Scientific).

A Biozen Oligo column (2.1 × 100 mm, 1.7 μm) (Phenomenex, Torrance, CA, USA) was used with the temperature held at 40°C. Mobile phase (A) consisted of water containing an amine/HFIP buffer, whereas mobile phase (B) was MeOH containing an amine/HFIP buffer. The amine concentration evaluated in the DoE study ranged from 4 to 10 mM, and the HFIP concentration from 40 to 60 mM. Gradient elution was as follows: 5% B at time = 0 min, increasing to 80% with different elution gradient slopes (between 2.5% and 5.3% B/min) in the DoE experiments. The flow rate was set at 0.20 mL min^−1^, and the injection volume was set at 2 μL. The optimised working conditions for FMV analysis were 4 mM DIPEA and 58 mM HFIP, with a gradient slope of 3.7% B/min. For TFR analysis, the optimised conditions were 7 mM of DIPEA and 52 mM of HFIP, with a gradient slope of 4.5% B/min.

The mass analysis was performed in ESI(−) mode, with potential at −2800 V, sheath gas at 60 units, auxiliary gas at 20 units, auxiliary gas temperature (AGT) at 400°C, capillary temperature at 300°C and stellarity lens (S‐lens) set at 90 V. The acquisition of data was in full MS mode, with a mass range extending from 550 to 2500 *m/z* throughout the run. The resolution was 70 000, and the MS AGC target was set at 1 × 10^6^. The source CID (sCID) was set at 0 V. The normalised collision energy (NCE) was set to 13, 15 and 17 V. The detailed chromatographic and mass acquisition parameters of the optimised conditions are fully reported in Table S1.

### 2.4 Software

Chromeleon Chromatography Data System (CDS) v.7.2 software (Thermo Fisher Scientific) was used to control LC and MS instruments, acquire data and analyse UV results. The LC‐MS data were processed using Xcalibur v.1.7 and BioPharma Finder v.5.3 software, with the ESI(−) mass spectra deconvolution performed at isotopic resolution using Xtract (all by Thermo Fisher Scientific). MODDE Pro Version 13.1 software (Sartorius Data Analytics, Göttingen, Germany) was used for planning the RSM experimental plan and for the related data treatment.

## 3 Results and Discussion

The variety of ON structures makes it difficult to choose a priori specific LC‐MS conditions, highlighting the need for comprehensive research to guide the selection of optimal methodologies. Additionally, efforts should be made to develop analytical procedures that can distinguish impurities within the parent ON and from each other, because a comprehensive and accurate depiction of the ON composition of the sample is essential to ensure the quality and safety of the API [4]. In view of the aforementioned issues, a systematic multivariate approach to development is recommended as also highlighted by recent ICH guidelines [37]. Although this approach is not yet mandatory, it has been emphasised that a scientifically based QC strategy for ONs should be encouraged [6, 9]. In this study, the analytical procedure development steps were as follows: (i) definition of the Analytical Target Profile (ATP) and Critical Method Attributes (CMAs); (ii) knowledge management; (iii) RSM; and (iv) definition of the individual sweet spots and optimum conditions for FMV and TFR analysis. The developed analytical procedures were then applied to characterise FMV and TFR impurities.

### 3.1 ATP and CMAs

The development of the analytical procedure depends on identifying the Critical Quality Attributes of the pharmaceutical product to be controlled. In this case, impurity profiling of the ASOs should be assessed to ensure the quality of the API. The ATP outlines the specific objectives of the analytical procedure [37] and consists of characterising FMV and TFR impurities and determining their percentage with respect to the parent compound, achieving reporting and identification thresholds of at least 0.2% and 1.0% respectively, as recently proposed [4, 5].

In order to achieve the ATP, the analytical technique was determined to be IP‐RP‐UHPLC‐HRMS ESI(−) due to its widely accepted status as the gold standard in the analysis of ONs. The IPR adsorbs to the stationary phase and allows for electrostatic interactions between the multiple negatively charged ONs, whereas non‐electrostatic interactions between the ONs and the alkyl/aryl chains of the stationary phase contribute to enhancing selectivity. The employment of negative ESI mode is particularly effective, given the polyanionic nature of these molecules. Conversely, positively charged cations of alkali metals are electrostatically attracted to the polyanionic backbone of ONs, which may result in the formation of adducts with Na^+^ and K^+^, leading in turn to a decline in both sensitivity and mass accuracy, because the available charge is distributed across the parent peak and its adducts [21].

Therefore, the CMAs selected for RSM study were related to both sensitivity and selectivity of the analytical procedure. With regard to the responses pertaining to sensitivity, initially both area and height were considered. However, the prosecution of the study revealed that the quality parameters of the calculated models by DoE, represented by coefficient of determination *R* ^2^ and goodness of prediction *Q* ^2^, were found to be better for peak heights in comparison to peak areas. Consequently, given the greater reliability of the models, it was decided that optimisation of peak height, rather than peak area, should be undertaken. Hence, for FMV analysis, the CMAs were represented by FMV peak height (h_F_), FMV baseline peak width (w_F_) and the resolution between FMV Impurity 1 and FMV Impurity 2 (R_12F_), which eluted sequentially in the chromatogram. For TFR analysis, the CMAs were selected as TFR peak height (h_T_), TFR baseline peak width (w_T_), the resolution between TFR Impurity 1 and TFR Impurity 2 (R_12T_) and the resolution between TFR Impurity 2 and TFR Impurity 3 (R_23T_). The FLP height and width values were obtained from the UV trace, whereas the resolution values were obtained from the extracted mass chromatograms.

### 3.2 Knowledge Management

The knowledge management process entails executing scouting experiments, with the objective of identifying the most appropriate analytical mode, including the stationary phase and the type of IPR, to facilitate the approach of the ATP. The selection of the ion‐pair system is determined by specific requirements, including compatibility with LC‐MS, sensitivity and the miscibility of IPRs and buffer components in the organic solvent [34].

#### 3.2.1 Stationary Phase and Mobile Phase

The selected column was Biozen Oligo (Phenomenex), which is designed for advanced ON analysis. This column is an organo‐silica, core–shell particle bonded with a C18 stationary phase. According to the manufacturer, it offers a unique combination of core–shell performance gains, such as speed, sensitivity and resolution, as well as the high pH ruggedness necessary for ON separations and inertness due to the titanium hardware.

The composition of the mobile phase influences both the chromatography and the MS data, including aspects such as sensitivity, charge state distribution and adduct ions [6]. The ON signal intensity is highly sequence dependent, but it is also greatly affected by the type and concentration of IPRs [28, 29, 30]. Depending on their hydrophobic/hydrophilic characteristics, alkylamines buffered with volatile acids have been classified as weak, moderate or strong IPRs. More hydrophilic IPRs are considered weak agents because they retain ONs less effectively and are favoured in applications where ONs differ in hydrophobicity, such as sequence variants or diastereoisomers. Conversely, more hydrophobic alkylamines act as strong IPRs and are typically employed for length‐based separations [34]. In this work, three alkylamines with different strength characteristics were selected, one for each class, to be tested and compared in terms of performance: TEA, as a weak IP agent; DIPEA, as a moderate IP agent; and DBA, as a strong IP agent. The counterion of the buffer system was selected as HFIP, due to its ideal chemical–physical properties for use in LC‐MS analysis, exhibiting a favourable combination of low acidity (pK_a_ = 7.98) and high volatility (boiling point = 59°C). In fact, HFIP not only adjusts the mobile phase pH, but also functions to be depleted early during droplet evaporation due to its volatility (unlike other commonly used acid modifiers such as acetic and formic acid). This process avoids concentration at the surface of the electrosprayed droplets and competition with ON ionisation. In addition, its acidity is adequate to provide enough protons to the negatively charged emitter, facilitating an efficient ESI process [29, 42]. Finally, the most frequently employed organic solvents in the mobile phase are MeOH or acetonitrile. In this study, MeOH was selected on the basis of its enhanced solubility in fluorinated alcohols [7] and its lower environmental impact.

#### 3.2.2 Scouting Phase

A preliminary scouting phase was conducted to select the most appropriate IPR type and main MS instrumental settings, that is, sheath gas, AGT and S‐lens, for FMV and TFR analysis. During this phase, the concentration of TEA and DBA was maintained at 5 mM, whereas the concentration of DIPEA was kept at 4 mM. The optimal values for MS parameters were determined based on the quality of the MS spectrum (charge state distribution, presence of adducts and in‐source fragments), the MS signal height and the chromatographic peak shape. In this step, mobile phase ageing was also considered, given that achieving stability in the mobile phases is one of the main challenges in ON analysis, necessitating daily preparation. The loss of MS sensitivity over brief periods may be associated with alkylamine oxidation and aggregate formation [43].

With regard to the MS parameters investigated, the sheath gas is employed to guide and stabilise the ion flow in the spectrometric system. The ionisation system functions as a ‘sheath’ around the ion flow, thereby reducing ion dispersion and facilitating their passage through the system. This, in turn, enhances the resolution and sensitivity of the system. AGT refers to the temperature of the auxiliary gas used to facilitate the evaporation of the sample. Finally, the S‐lens is found at the beginning of the ion‐transfer stage, particularly in the mass spectrometers that use ionisation at atmospheric pressure. The primary function of the ion guide is to concentrate the ions generated in the ionisation source, thereby facilitating their efficient transportation to the detector. This enhancement in transmission efficiency serves to mitigate ion dispersion, minimising the energy dispersed during the ionisation process [44].

The experimental parameters and the results of the scouting experiments are reported in Table S2 for both FMV and TFR analyses. These include quantitative information on MS peak height and qualitative information on MS spectrum quality. The quantitative results are visualised in the bar graphs reported in Figure S1a for FMV and in Figure S1b for TFR. Experiments E1–E6 were run with TEA (blue bars), E7–E13 with DBA (orange bars) and E14–E15 with DIPEA (green bars). Examples of the obtained MS spectra are shown in Figure S2 for FMV and in Figure S3 for TFR. These preliminary experiments clearly indicated that DIPEA emerged as the most efficacious option among the three alkylamines. Furthermore, prior experimentation with TEA and DBA yielded valuable insights into the effects of the other MS parameters. Consequently, E14–E15 outcomes were deemed sufficiently informative for selecting DIPEA.

##### 3.2.2.1 Fomivirsen: Selection of Alkylamine and MS Parameters

The effects of parameter changes on MS peak height of FMV are visualised in Figure S1a. The employment of TEA in Experiment E2, which utilised a sheath gas of 60 units, resulted in a more intense signal than Experiment E1; anyway, increasing AGT from 320°C to 400°C in Experiment E3 produced an even more pronounced effect, resulting in a 16‐fold increase in intensity. The maximum MS peak height was obtained in Experiment E4, where S‐lens was increased from 60 to 90 V and AGT was set at 400°C, whereas at higher temperatures of the auxiliary gas (Experiments E5–E6), the signal decreased. Chromatographically, the use of TEA resulted in poor FMV retention (retention time about 5.9 min) and significant peak tailing, whereas the ESI mass spectrum did not show significant adduct ions nor in‐source fragments; the charge state distribution envelope was regular, with the eight‐charged species being the most abundant (Figure S2a). The optimal instrumental conditions (sheath gas, 60 units; AGT, 400°C; S‐lens, 90 V) were adopted for subsequent analyses.

The utilisation of DBA as alkylamine yielded an MS signal around one half lower than the best TEA result (Figure S1a), but better chromatographic retention (retention time about 10.6 min) with a sharp and symmetric peak (Figure S2b). The change in the spectrum appearance was remarkable, with the highest charge state shifted to four. Moreover, the presence of stable adducts, made of up to five DBA molecules per FMV charge state, complicated the interpretation of the resulting spectrum, besides being responsible for the charge state reduction and the MS signal lowering due to partial neutralisation of the original ON negative charge. A series of experiments (E10–E13) were conducted to ascertain the combined effect of varying the AGT values at 450°C and 500°C, both with and without sCID, in order to promote the adduct dissociation. However, this resulted in some in‐source fragmentation and did not lead to a significant improvement in results.

The most favourable outcomes were achieved with DIPEA, which resulted in a signal intensity enhancement of approximately 100% in comparison with TEA (Figure S1a). The chromatographic peak was sharp and symmetric (retention time about 6.9 min), whereas the mass spectrum was found to be free of adduct ions and exhibited a high degree of similarity to that obtained with TEA (Figure S2c). The analytes' stability in the autosampler at 4°C was evaluated for 24 h through seven replicates of Experiment E15, resulting in no significant changes in peak areas nor in impurity profile. The RSD of the FLP extracted MS chromatogram peak area for FMV was 1.7%.

##### 3.2.2.2 Tofersen: Selection of Alkylamine and MS Parameters

The results of the above investigation on FMV, when performed on TFR, were essentially the same. With regard to the experiments analysing TFR with TEA as IPR (Figure S1b), the increase of sheath gas value from 40 to 60 units (Experiments E1–E2) induced a moderate decrease in signal intensity. In Experiment E3, the AGT value increased from 320°C to 400°C. This manipulation produced a considerably more pronounced effect, leading to an approximately threefold increase in intensity overall from Experiment E1 to Experiments E3–E4. The results of Experiments E5–E6 demonstrated that an increase in AGT to values above 400°C resulted in a moderate decline in signal intensity.

As observed for FMV, substituting TEA for DBA in Experiments E7–E13 was found to significantly impact the retention time, increasing it from about 6.2 to 10.7 min and decreasing the peak height overall (Figures S1b and S3a,b). Concurrently, the ESI‐MS spectrum underwent substantial alteration, with the emergence of peaks resulting from the formation of oligonucleotide‐DBA adducts, and a shift in charge state distribution towards lower values. To reduce the formation of adducts, tests were carried out using sCID values of 5 and 10 V. Increasing this parameter was found to reduce the formation of adducts to some extent. However, when sCID was set to 10 V (Experiment E8), other peaks emerged that indicated source degradation of the analytes. Although an increase in AGT has been shown to reduce adduct formation (Figure S3b), the results obtained using DBA were generally of lower quality than those obtained using TEA. Consequently, DBA was discarded.

Conversely, the use of DIPEA produced a 75% increase in the TFR signal intensity compared to TEA (Experiments E14–E15) while maintaining a sharp and symmetric chromatographic peak and an acceptable retention (retention time 7.4 min), as well as a good mass spectral quality (Figure S3c). Setting sCID at 5 V did not lead to a significant improvement in results. TFR was also found to be stable at 4°C for at least 24 h. The RSD of the FLP peak area was 2.5% after seven replicates of Experiment E15.

Based on the results of testing the FMV and TFR samples, DIPEA was selected as IPR, and the same instrumental MS conditions were fixed for the analysis of both ASOs: a sheath gas value of 60 units, an AGT value of 400°C and an S‐lens value of 90 V.

### 3.3 RSM

#### 3.3.1 Selection of Experimental Domain, Models and Experimental Plan

Following the preliminary phase experiments for the selection of DIPEA and the fundamental MS conditions, further experiments were conducted using a wide range of DIPEA and HFIP concentrations to prepare the mobile phase, in order to identify an appropriate experimental domain for the RSM step.

Initially, the mobile phase contained 4 mM DIPEA, 12.5 mM HFIP, with a gradient slope of 5.3% B/min. Subsequently, the concentration of HFIP was first increased to 25 mM and then to 50 mM, whereas the DIPEA concentration was increased from 4 to 8 mM and then to 80 mM. The latter value was chosen based on a significant decrease in retention in the 20–100 mM concentration range of the IP agent, as reported by Sutton et al. [33], compared to lower values. Increasing the DIPEA concentration to 8 mM improved the peak shape and intensity for both FMV and TFR; however, increasing it to 80 mM caused a marked deterioration in peak shape for both. The lower value of the experimental domain for DIPEA concentration was set at 4 mM, in accordance with the literature findings, to avoid a drastic reduction of MS signal [42].

Additionally, the elution gradient was evaluated as a third factor, with values set at 2.5% and 3.5% B/min. For both ASOs, these gradient values substantially improved the peak shape and completely eliminated the initial tailing. The application of a gradient of 3.5% B/min produced optimal outcomes for FMV, yielding a more symmetric peak. Conversely, TFR exhibited a distinct pattern, with both slopes producing two peaks of similar quality.

Based on these results, RSM was directly implemented to conduct a more in‐depth investigation within the following intervals: DIPEA concentration (*DIPEA conc*.), 4–10 mM; HFIP concentration (*HFIP conc*.), 40–60 mM; gradient slope (*slope*), 2.5%–4.5% B/min. RSM was used to optimise the chromatographic responses, providing a comprehensive evaluation of the effectiveness of the chromatographic conditions in terms of sensitivity and selectivity. The selected responses (CMAs) were the height and width of FMV and TFR main peaks in the UV chromatogram, as well as the resolutions involving selected impurity peaks. As a matter of fact, it was regarded as sufficient to optimise the parameters influencing the MS response through scouting exploratory studies. On the other hand, UV optimisation was deemed to deserve more attention by a multivariate approach, due to its intrinsic lower sensitivity and its widespread use in quantitative applications.

The model used to correlate factors and responses was a quadratic polynomial, the equation of which is provided below:

$$\begin{array}{c} y=\beta_{0}+\beta_{1}x_{1}+\beta_{2}x_{2}+\beta_{3}x_{3}+\beta_{11}{x_{1}}^{2}+\beta_{22}{x_{2}}^{2}\\ \;+\beta_{33}{x_{3}}^{2}+\beta_{12}x_{1}x_{2}+\beta_{13}x_{1}x_{3}+\beta_{23}x_{2}x_{3}+\varepsilon \end{array}$$

In this model, the response variable is denoted *y*, whereas the independent variables are indicated as *x₁* (*DIPEA conc*.), *x*_*2*_ (*HFIP conc*.) and *x₃* (*slope*). The intercept is represented by *β₀*; the linear, quadratic and interaction terms are represented by *β*_*i*_, *β*_*ii*_ and *β*_*ij*_, respectively; and the experimental error is represented by ε. Box–Behnken design was used to calculate the model's coefficients. In this type of design, all factors are investigated at three levels, and combinations of factors exhibiting extreme values for all three factors are excluded [36]. The experimental plan consisted of 15 experiments, including three centre‐point runs for the estimation of the experimental variance. The experiments were conducted in a randomised order.

#### 3.3.2 Fomivirsen Contour Plot Analysis

In the FMV analysis, the following CMAs were considered: the FMV peak height (h_F_), the FMV baseline peak width (w_F_) and the resolution between FMV Impurity 1 and FMV Impurity 2 (R_12F_). The exact mass of FMV Impurity 1 was found to be 6357.561 Da, which can be attributed to the loss of a deoxyribose‐thymine phosphorothioate nucleotide. FMV Impurity 2 had an exact mass of 6661.619 Da, which can be attributed to the conversion of a sulphur atom to an oxygen atom (see Section 3.4.1).

The experimental plan is reported in Table 1 with the measured responses. It should be observed that the chromatographic resolution values are extremely low for FMV, from less than 0.100 to 0.560, and similar results were obtained with TEA and DBA. This means that all the peaks are essentially unresolved under all the tested conditions. This is obviously an issue for UV detection, but not for high‐resolution MS, whose selectivity allows discrimination of coeluting compounds with very close molecular masses. Given the case‐study character of this work, no further attempts were made to improve selectivity by using alternative IP agents and fluoroalcohols or different separation techniques.

**TABLE 1:** RSM: Box–Behnken design and responses.

Exp. no.	DIPEA concentration (mM)	HFIP concentration (mM)	Gradient slope (%B/min)	Fomivirsen			Tofersen			
				h_F_ (counts)	w_F_ (min)	R_12F_	h_T_ (counts)	w_T_ (min)	R_12T_	R_23T_
1	4	40	3.5	229.10	0.117	0.316	142.68	0.092	0.705	0.298
2	10	40	3.5	214.20	0.127	0.048	128.46	0.102	0.758	0.115
3	4	60	3.5	286.82	0.085	0.261	135.77	0.089	0.955	0.516
4	10	60	3.5	248.00	0.104	0.178	134.16	0.096	0.840	0.229
5	4	50	2.5	198.92	0.126	0.416	117.38	0.108	0.995	0.492
6	10	50	2.5	176.33	0.144	0.072	109.64	0.113	1.119	0.158
7	4	50	4.5	288.81	0.082	0.194	143.32	0.081	0.684	0.295
8	10	50	4.5	272.31	0.093	0.097	151.78	0.080	0.727	0.161
9	7	40	2.5	173.48	0.158	0.113	104.10	0.128	0.793	0.262
10	7	60	2.5	211.38	0.121	0.560	118.30	0.110	1.085	0.427
11	7	40	4.5	274.11	0.098	0.082	160.62	0.080	0.589	0.100
12	7	60	4.5	280.69	0.085	0.067	144.43	0.082	0.675	0.597
13	7	50	3.5	221.13	0.105	0.314	122.61	0.093	0.813	0.318
14	7	50	3.5	266.20	0.097	0.197	137.37	0.094	0.907	0.278
15	7	50	3.5	269.09	0.096	0.136	140.52	0.092	0.888	0.344

Abbreviations: DIPEA, *N*,*N*‐diisopropylethylamine; h_F_, FMV peak height; HFIP, 1,1,1,3,3,3‐hexafluoroisopropanol; h_T_, TFR peak height; R_12F_, resolution FMV Impurity 1/FMV Impurity 2; R_12T_, resolution TFR Impurity 1/TFR Impurity 2; R_23T_, resolution TFR Impurity 2/TFR Impurity 3; w_F_, FMV baseline peak width; w_T_, TFR baseline peak width.

The models were subjected to refining by removing non‐significant terms to improve their predictive power. ANOVA showed that they were all valid and significant (Table S3), with satisfactory values of *R* ^2^ and *Q* ^2^ (Table S4) [36]. The observed versus predicted plot is displayed in Figure S4. Consequently, these models could be used for optimisation through examination of graphical analysis of effects and contour plots.

The graphical analysis of effects is presented in Figure S5, providing information on the weight and sign of the coefficients of the models for the different responses. The length of the bar indicates the weight of the effect, and the error bars represent the confidence interval. The term with the greatest impact on h_F_ is the positive linear term relative to *slope*, with the linear term *HFIP conc*. also exerting a significant positive effect (Figure S5a). Regarding w_F_ (Figure S5b), there are significant negative linear effects of *HFIP conc*. and *slope*, whereas a positive linear term is evidenced for *DIPEA conc*. Also, significant quadratic effects are detected, one for *HFIP conc*. and one for *slope*, as well as a positive interaction effect between these two factors. Finally, both *DIPEA conc*. and *slope* exert negative linear effects on R_12F_, on which the interaction between *HFIP conc*. and *slope* is also significant (Figure S5c).

**FIGURE 2:**
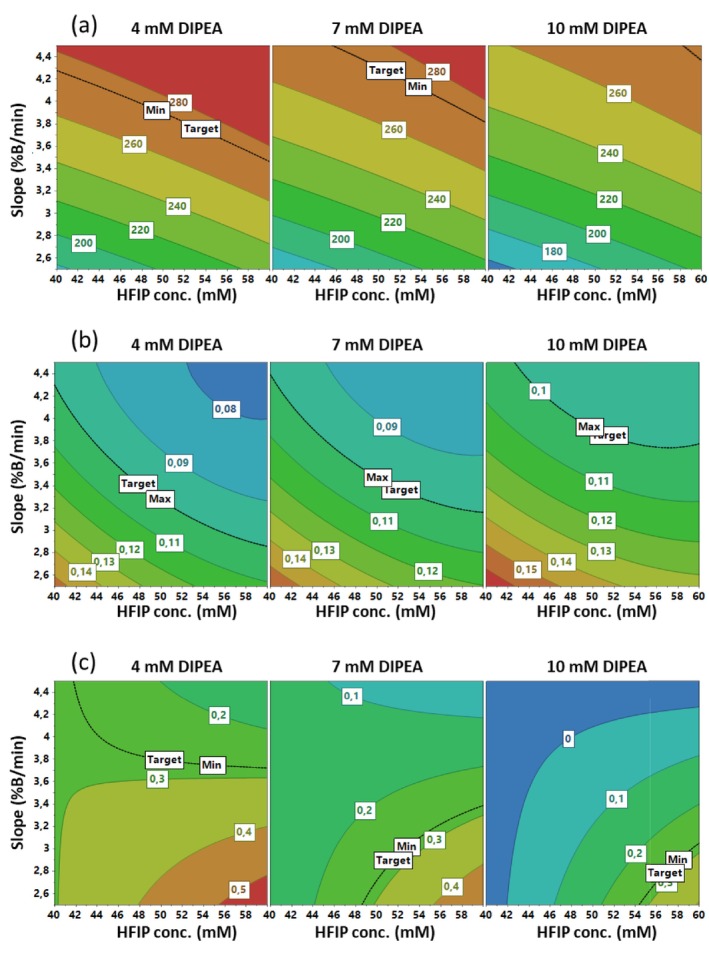
Box–Behnken contour plots obtained by plotting *slope* versus *HFIP conc.* at three different values of *DIPEA conc.* (4, 7 and 10 mM). (a) FMV peak height (h_F_), (b) FMV baseline peak width (w_T_) and (c) R_12T_ (resolution FMV Impurity 1/FMV Impurity 2).

Subsequently, contour plots were drawn (Figure 2), and a map of the predicted responses was visualised, by plotting *slope* versus *HFIP conc*. at three distinct levels of *DIPEA conc*. (4, 7 and 10 mM). h_F_ contour plots (Figure 2a) show that height maximisation is achieved at low *DIPEA conc*. levels, high *slope* levels and high *HFIP conc*. levels. Notably, no curvature is observed in the model. In the plot where *DIPEA conc*. is set at 4 mM, the optimal height is observed over a broad range, whereas in the plot where this factor is maintained at 10 mM, a decrease in the maximum attainable height is evident. Considerable quadratic effects in the w_F_ model are observed from the curvatures detected in the related contour plots (Figure 2b). This response is minimised at elevated *HFIP conc*. and *slope* and reduced *DIPEA conc*. values. Finally, with regard to R_12F_, maximisation is obtained in the red zone, at low *DIPEA conc*. and *slope* levels and at high *HFIP conc*. (Figure 2c). The presence of a negative interaction between *HFIP conc*. and *slope* is also evident.

#### 3.3.3 Tofersen Contour Plot Analysis

For TFR analysis, the CMAs selected were TFR peak height (h_T_) and TFR baseline peak width (w_T_), as well as the resolutions between TFR Impurity 1/TFR Impurity 2 (R_12T_) and between TFR Impurity 2 and TFR Impurity 3 (R_23T_). Exact masses of TFR Impurity 1, TFR Impurity 2 and TFR Impurity 3 were determined to be 6352.009 Da (deletion of 2′‐methoxyethylribose‐5‐methylcytosine phosphate and 2′‐methoxyethylribose‐5‐methyluridine phosphorothioate), 6730.095 Da (deletion of 2′‐methoxyethylribose‐5‐methylcytosine phosphorothioate) and 7107.184 Da (sulphur atom to oxygen atom), respectively (see Section 3.4.2). The Box–Behnken experimental plan, along with the responses obtained, is presented in Table 1.

The models were refined and were found valid and significant according to ANOVA, except for the w_T_ response, for which the model was not valid (Table S3). Nevertheless, the quality parameters of the models, including *R* ^2^ and *Q* ^2^, were good for all responses, as shown in Table S4 [36]. The observed versus predicted plot is shown in Figure S6.

The graphical analysis of the effects is shown in Figure S7. As can be seen in Figure S7a, *slope* exerts a significant positive effect on h_T_, whereas the only other significant coefficient is the interaction between *HFIP conc*. and *slope*, but its impact is much lower. *Slope* also exerts an overwhelming negative effect on w_T_ (Figure S7b), indicating that high values of this factor are favourable for optimising both sensitivity and peak shape. A positive linear effect of *HFIP conc*. and a negative linear effect of *slope* are observed on R_12T_, as well as a negative quadratic effect of *HFIP conc*. (Figure S7c). Finally, Figure S7d shows a negative linear effect of *DIPEA conc*. on R_23T_, but a positive linear effect of *HFIP conc*. is evident. Furthermore, a positive interaction can be observed between *HFIP conc*. and *slope*.

**FIGURE 3:**
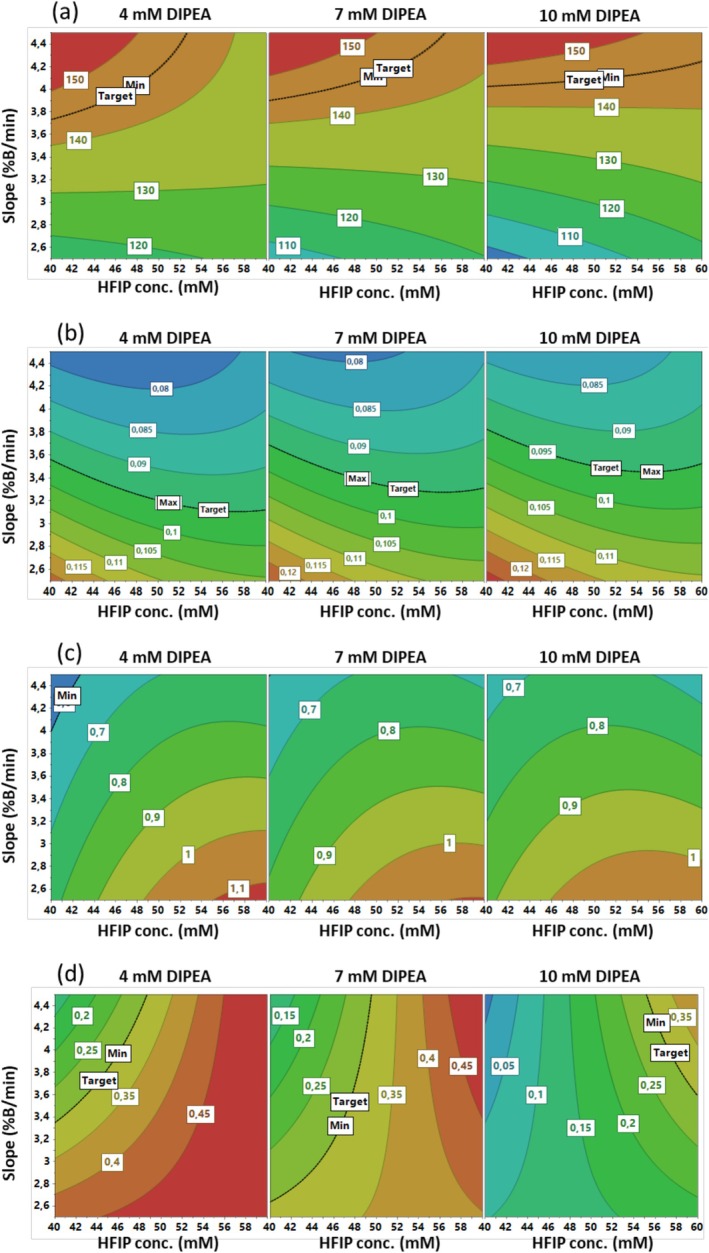
Box–Behnken contour plots obtained by plotting *slope* versus *HFIP conc.* at three different values of *DIPEA conc.* (4, 7 and 10 mM). (a) TFR peak height (h_T_), (b) TFR baseline peak width (w_T_), (c) R_12T_ (resolution TFR impurity 1/TFR Impurity 2) and (d) R2_3T_ (resolution TFR impurity 2/TRF Impurity 3).

With regard to the h_T_ contour plot (Figure 3a), it is evident that optimisation is attained in the red zone, at low values of *HFIP conc*. and high values of *slope*, in accordance with the highlighted interaction between these two factors. The pattern of the isoresponse curves is similar for the different plotted values of *DIPEA conc*. (4, 7 and 10 mM). The minimisation of w_T_ (Figure 3b) is achieved by setting a high *slope* value and a low *DIPEA conc*. value. The blue area, where the predicted values for w_T_ are equal to or less than 0.08, is larger for *DIPEA conc*. at 4 mM than at 10 mM. An examination of the R_12T_ contour plots (Figure 3c) reveals the curvature resulting from the quadratic effect of *HFIP conc*. The maximisation of this response is achieved in the red‐orange zone, which is characterised by medium‐high *HFIP conc*. values and low *slope* levels. R_23T_ is the sole response on which *DIPEA conc*. exerts a significant effect (Figure 3d). The highest predicted values of this response, which are located in the red zone, are observed at low levels of *DIPEA conc*. and high levels of *HFIP conc*. For this response, an interaction between *HFIP conc*. and *slope* is also observed. In particular, an interesting result is obtained when *DIPEA conc*. is utilised at 4 mM: the maximum resolution value of 0.45 is recorded for a range of *HFIP conc*. from 45 to 60 mM and over the entire range of *slope* values studied (from 2.5% to 4.5% B/min).

#### 3.3.4 Sweet Spot Plot and Optimised Method Conditions

**FIGURE 4:**
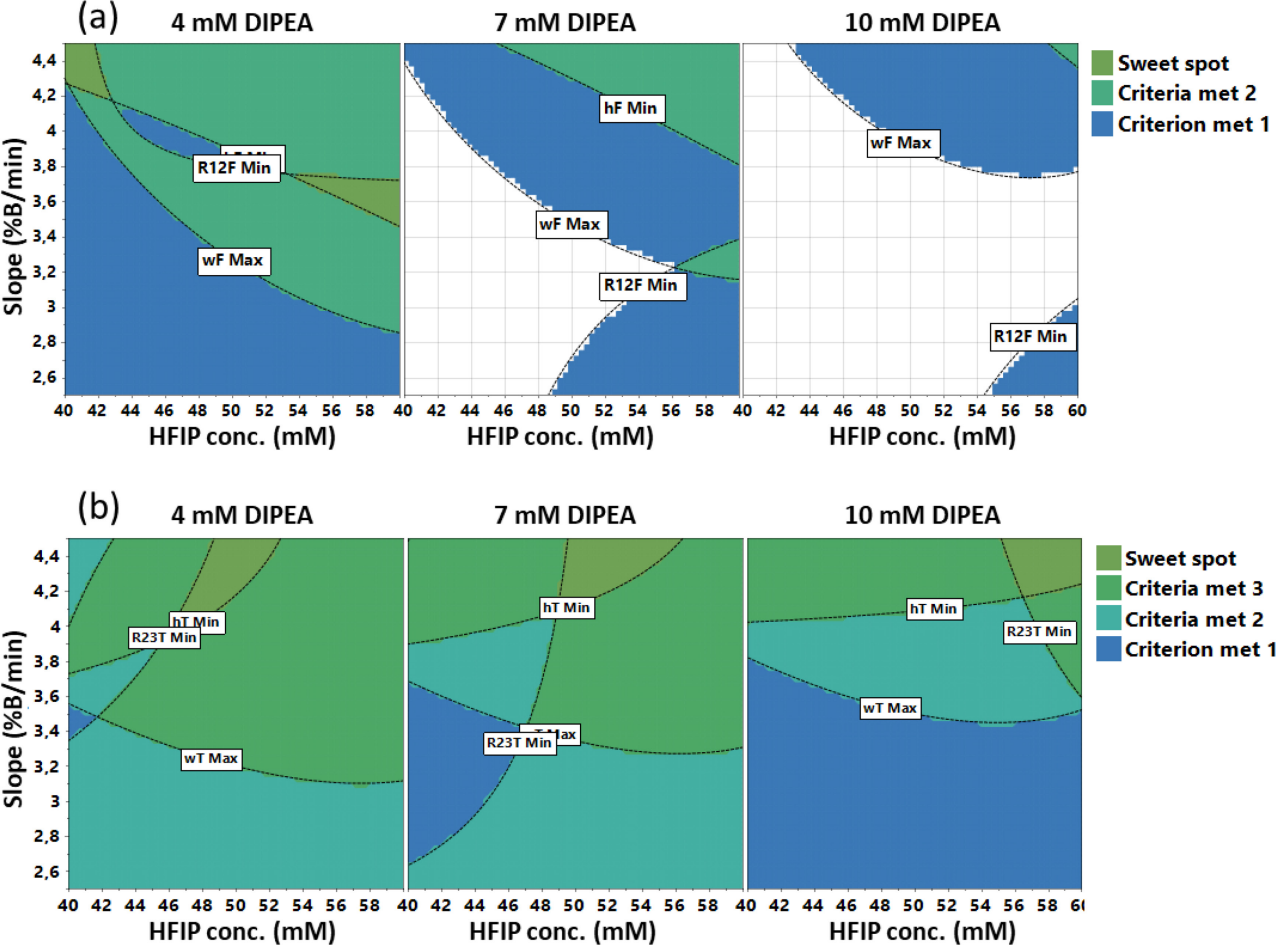
Sweet spot plots obtained by plotting *slope* versus *HFIP conc.* at three different values of *DIPEA conc.* (4, 7 and 10 mM). (a) Fomivirsen, (b) Tofersen.

The information obtained from the contour plots was synthesised by superimposing the predicted values for the responses, thus obtaining the sweet spot plot. This is a graph where it is possible to visualise the areas where the targets for the CMAs are simultaneously met. The sweet spot plots obtained for FMV and TFR are reported in Figure 4. In order to conduct FMV analysis, the following values were set as the targets for the responses: h_F_, 275 counts; w_F_, 0.100 min; R_12F_, 0.250. For the purpose of TFR analysis, the response targets were defined as follows: h_T_, 145 counts; w_T_, 0.095 min; R_12T_, 0.650; R_23T_, 0.330. The “sweet spot” is defined as the dark green zone, where all requirements for all the responses are met. Other colours highlight zones where fewer or no criteria are met, as illustrated in Figure 4.

The choice of the target values was driven by the following considerations, which were mostly based on the data reported in Table S5, including RSM statistics of the measured responses. Firstly, it was necessary to find a compromise between sensitivity and selectivity, considering that a high level of impurity separation was not required due to the power of HRMS, whereas the increase of peak height and the decrease of peak width were fundamentally to be obtained by DoE. Secondly, the median of the RSM measured values for each response was used as a starting point in the identification of the target for enabling the establishment of a minimum requisite reasonably achievable. Thirdly, a comparative analysis of the measured resolutions was conducted, which revealed that R_12T_ generally exhibited higher values compared with R_23T_, underscoring the necessity to prioritise the enhancement of the latter. Moreover, it is worthwhile to note that no separation method, chromatographic or electrophoretic, is to date available to fully resolve the ON impurities. In this regard, the scope of the resolution optimisation was to minimise the risk of signal suppression for impurities with retention time close to the FLP, as well as to avoid to miss, even by HRMS, an impurity with a retention time and mass very close to another one. On this respect, the resolution performances requested, even if far from a satisfactory chromatographic separation, were deemed suitable for the analytical aim.

The FMV sweet spot plot is shown in Figure 4a. It is evident that the presence of the dark green zone is conditional upon the *DIPEA conc*. being fixed at 4 mM; conversely, in the 10 mM plot, a substantial white zone is observed, indicating an absence of target fulfilment. In the case of the TFR sweet spot plot, the dark green zone was observed to be situated at high values of slope, with diverse values for the other two factors (Figure 4b).

**FIGURE 5:**
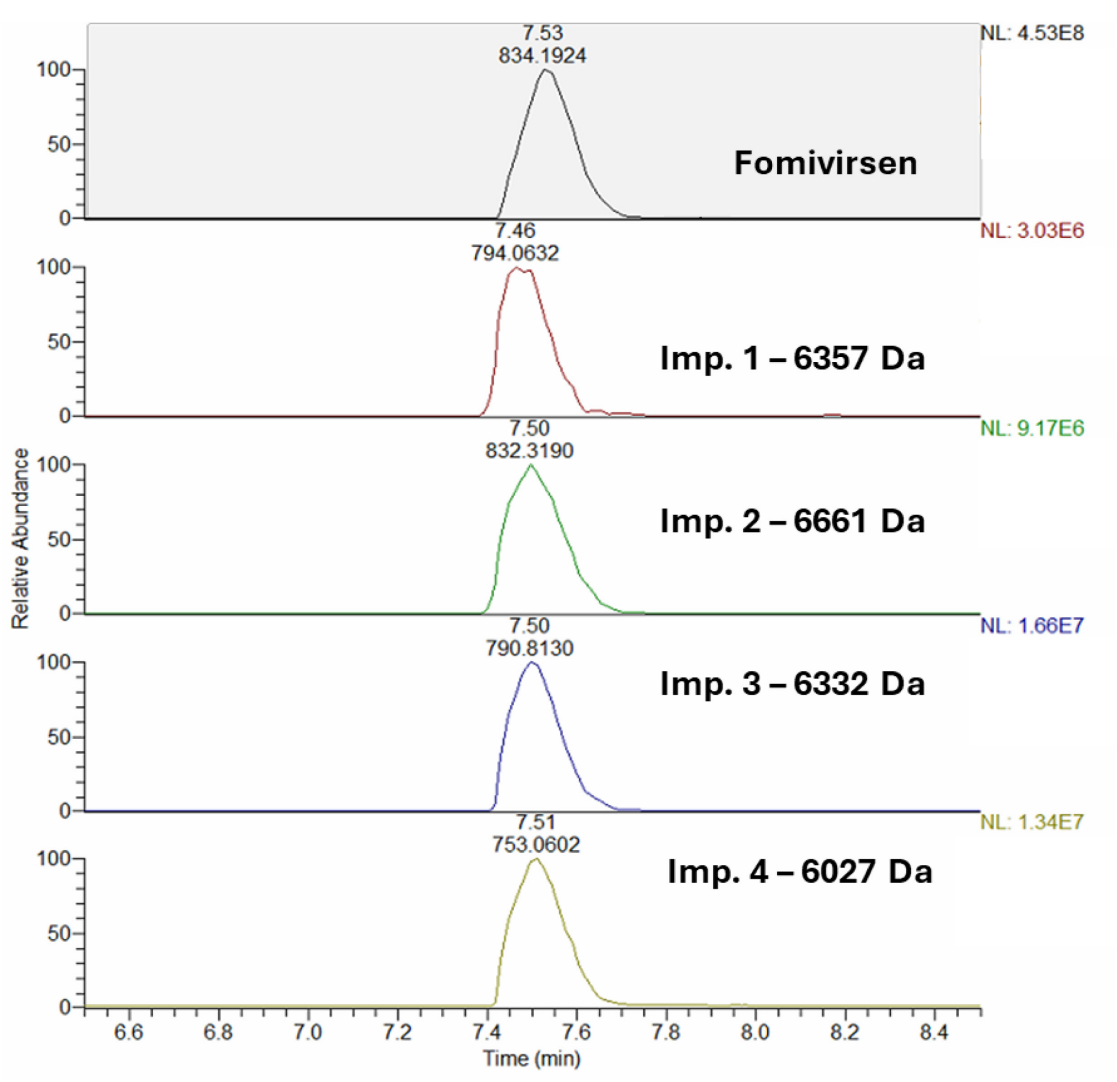
Extracted mass chromatograms of fomivirsen and Impurities 1–4.

**FIGURE 6:**
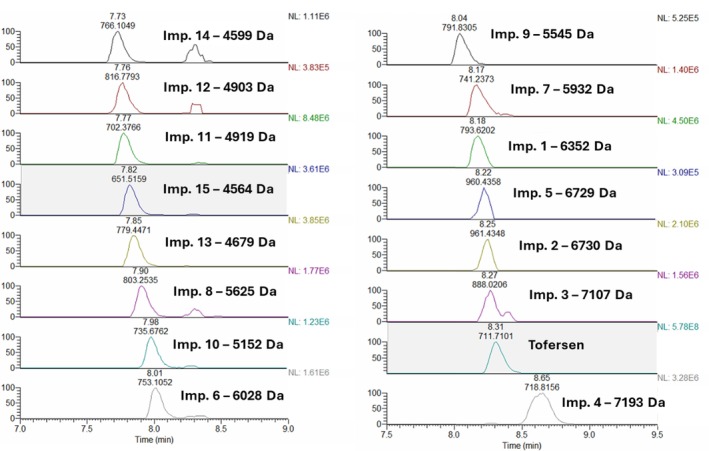
Extracted mass chromatograms of tofersen and Impurities 1–15.

The optimised conditions corresponded to the following: for FMV, 4 mM DIPEA, 58 mM HFIP and 3.7% B/min; for TFR, 7 mM DIPEA, 52 mM HFIP and 4.5% B/min. Applying these conditions, general agreement was found between the predicted and measured responses (Table S5). The high‐resolution extracted mass chromatograms of the FLPs and their impurities obtained in the optimised conditions are shown in Figures 5 and 6 for FMV and TFR, respectively. As can be observed in Figure 6, certain extracted MS impurity traces exhibit the presence of second peaks. This is particularly evident in the case of Impurity 14 and Impurity 12. These peaks have the same retention time as the FLP (8.30 min) and are characterised by irregularity and distortion in shape. These observations suggest that these peaks are likely artefacts arising from minor interferences induced by the overwhelming amount of coeluting FLP. In fact, peak distortion does not occur in the case of true coeluting impurities, such as TFR Impurity 2 and FMV impurities (Figure 5). The only probable exception may be TFR Impurity 3, which shows a smaller, poorly separated peak at higher retention time. Alternative explanations could be proposed for the latter, such as the presence of a positional isomer.

### 3.4 Impurities Characterisation

The optimised conditions were successfully employed to characterise FMV and TFR impurities, which are fully described in Tables 2 and 3, respectively.

**TABLE 2:** Characterisation of fomivirsen impurities.

Compound	Description	Monoisotopic mass (mean)	Theoretical mass (Da)	Matched mass error (ppm)	Fractional abundance (%)	Retention time (min)
Fomivirsen	Gd‐sCd‐sGd‐sTd‐sTd‐sTd‐sGd‐sCd‐sTd‐sCd‐sTd‐sTd‐sCd‐sTd‐sTd‐sCd‐sTd‐sTd‐sGd‐sCd‐sGd	6677.599	6677.589	1.6	96.59 (*0.50*)	7.53 (*0.12*)
Impurity 1	Deletion of sTd (*n* − 1)	6357.561	6357.565	−0.7	0.23 (*30.01*)	7.44 (*0.06*)
Impurity 2	Phosphorothioate to Phosphate	6661.619	6661.611	1.1	0.53 (*11.95*)	7.47 (*0.05*)
Impurity 3	Deletion of sGd (*n* − 1)	6332.561	6332.559	0.3	1.46 (*21.20*)	7.51 (*0.09*)
Impurity 4	Deletion of sGd‐sCd (*n* − 2)	6027.532	6027.535	−0.5	1.19 (*18.18*)	7.48 (*0.09*)

*Note:* Average data (*n* = 2). %RSD values of fractional abundance and retention time are reported in parentheses.

**TABLE 3:** Characterisation of tofersen impurities.

Compound	Description	Monoisotopic mass (mean)	Theoretical mass (Da)	Matched mass error(ppm)	Fractional abundance (%)	Retention time (min)
Tofersen	Mw‐sAw‐pGw‐sGw‐pAw‐sTd‐sAd‐sMd‐sAd‐sTd‐sTd‐sTd‐sMd‐sTd‐sAd‐sMw‐pAw‐sGw‐pMw‐sVw	7123.170	7123.159	1.5	93.91 (*2.06*)	8.30 (*0.06*)
Impurity 1	Deletion of pMw‐sVw	6352.007	6352.000	1.1	0.60 (*0.31*)	8.17 (*0.06*)
Impurity 2	Deletion of sMw	6730.096	6730.096	0.0	0.49 (*11.75*)	8.24 (*0.02*)
Impurity 3	Phosphorothioate to Phosphate	7107.182	7107.182	0.0	0.90 (*12.91*)	8.26 (*0.03*)
Impurity 4	+ Isobutyryl (incomplete deprotection of G)	7193.221	7193.201	2.9	0.50 (*14.53*)	8.66 (*0.13*)
Impurity 5	Deletion of sVw	6729.101	6729.099	0.2	0.21 (*1.75*)	8.20 (*0.02*)
Impurity 6	Deletion of Gw‐pMw‐sVw	6028.889	6028.877	1.9	0.16 (*0.07*)	8.01 (*0.04*)
Impurity 7	Deletion of sGw‐pMw‐sVw	5932.940	5932.934	1.0	0.18 (*1.38*)	8.16 (*0.06*)
Impurity 8	Deletion of Aw‐sGw‐pMw‐sVw	5625.813	5625.806	1.2	0.22 (*3.10*)	7.90 (*0.01*)
Impurity 9	Deletion of pAw‐sGw‐pMw‐sVw	5545.847	5545.839	1.5	0.19 (*1.34*)	8.05 (*0.04*)
Impurity 10	Deletion of sMw‐pAw‐sGw‐pMw‐sVw	5152.771	5152.763	1.5	0.22 (*0.12*)	7.97 (*0.11*)
Impurity 11	Deletion of Ad‐sMw‐pAw‐sGw‐pMw‐sVw	4919.678	4919.672	1.2	1.02 (*1.62*)	7.78 (*0.12*)
Impurity 12	Mw‐sAw‐pGw‐sGw‐pAw‐sTd‐sAd‐sMd‐sAd‐sTd‐sTd‐sTd‐sMd‐pTd	4903.703	4903.695	1.7	0.10 (*1.13*)	7.76 (*0.07*)
Impurity 13	Unknown	4679.715	—	—	0.55 (*1.49*)	7.84 (*0.03*)
Impurity 14	Deletion of Td‐sAd‐sMw‐pAw‐sGw‐pMw‐sVw	4599.657	4599.649	1.7	0.14 (*0.38*)	7.72 (*0.08*)
Impurity 15	Unknown	4564.653	—	—	0.59 (*0.71*)	7.81 (*0.08*)

*Note:* Average data (*n* = 2). %RSD values of fractional abundance and retention time are reported in parentheses.

#### 3.4.1 Fomivirsen Impurities

Four impurities above 0.1% were detected in FMV, all of which have been identified based on their exact mass difference with the FLP (6677.599 Da).

Impurity 1 has an observed mass of 6357.561 Da. The mass difference of −320.038 Da corresponds to the deletion of one deoxyribose‐thymine phosphorothioate (sTd), which has a theoretical mass of 6357.565 Da. In the intact oligonucleotide sequence, there are 10 such nucleotides. The observed peak is most probably an unseparated mixture of impurities with randomly distributed deletion positions. Under the optimised conditions, this peak has a retention time of 7.44 min and an observed relative abundance of 0.23%, as returned by the BioPharma Finder software. It has to be pointed out that, as with all the other impurities described in both model ONs, full validation of their relative amount is outside the scope of this work. However, it is noteworthy that this approach can accurately measure the monoisotopic accurate masses and identify impurities at a relative amount well below the currently accepted identification threshold of 1% [5].

Impurity 2 has a mass of 6661.619 Da, which is 15.980 Da lower than FMV. This value corresponds to the conversion of an S atom to an O atom in a phosphorothioate group, at an undefined position (theoretical mass of 6661.611 Da). As in the previous case, the chromatographic peak, with a retention time of 7.47 min and a relative abundance of 0.53%, is probably composed of a number of isomeric impurities involving the conversion of phosphorothioate to phosphate at different nucleotides.

Impurity 3 has a mass of 6332.561 Da, corresponding to the loss of a deoxyribose‐guanine‐phosphorothioate (sGd; exact mass: 6332.559 Da). The occurrence of only three such nucleotides in the FMV sequence, as well as Impurity 4 (see below), clearly deriving from the loss of the 3′ end dinucleotide, which contains sGd, allows us to postulate that Impurity 3 is prevalently formed by the deletion of the 3′‐terminal sGd. Under the optimised conditions, its retention time and relative abundance are 7.51 min and 1.46%, respectively.

Impurity 4 belongs to the *n*‐2 category: Its mass is 6027.532 Da, corresponding to the deletion of the 3′‐terminal deoxyribose‐cytosine phosphorothioate‐deoxyribose‐guanine phosphorothioate dinucleotide (theoretical mass of 6027.535 Da). It has a retention time of 7.48 min and a relative abundance of 1.19%.

#### 3.4.2 Tofersen Impurities

Fifteen impurities above 0.1% were detected in TFR, 13 of which were successfully identified. Through detailed observation of the sequence, it was deduced that the impurities are predominantly attributable to losses of nucleotides from the 3′ end. The observed monoisotopic mass of TFR is 7123.170 Da (theoretical value: 7123.159 Da).

Molecular mass of Impurity 1 is 6352.007 Da, which is in close accordance with the deletion of the 3′‐terminal dinucleotide 2′‐methoxyethylribose‐5‐methylcytosine phosphate‐2′‐methoxyethylribose‐5‐methyluracile phosphorothioate (pMw‐sVw, theoretical mass of 6352.000 Da). Under the optimised conditions, the retention time is 8.17 min, and the relative abundance is 0.60%.

Impurity 2 has a molecular mass of 6730.096 Da, with a mass difference of −393.074 Da with respect to TFR. This corresponds to the deletion of 2′‐methoxyethylribose‐5‐methylcytosine phosphorothioate (sMw, theoretical mass of 6730.096 Da). The optimised retention time and the percent abundance are 8.24 min and 0.49%, respectively.

The mass of Impurity 3 is 7107.182 Da, which is consistent with the conversion of a phosphorothioate to a phosphate, at an undefined sequence position (theoretical value: 7107.182 Da). The retention time is 8.26 min, and the percent abundance is 0.90%.

Impurity 4 has a monoisotopic mass of 7193.221 Da, with a mass difference of +70.051 Da compared to TFR. This indicates the addition of an isobutyryl group to guanine, resulting from its incomplete deprotection [7] (theoretical mass: 7193.201 Da). Given its lower polarity, this impurity is chromatographically separated from TFR, with a retention time of 8.66 min. Its relative abundance is 0.50%.

Impurity 5 has a mass of 6729.101 Da and a retention time of 8.20 min. The mass difference from the FLP, −394.069 Da, is in agreement with the deletion of the nucleotide at the 3′ end, 2′‐methoxyethylribose‐5‐methyluracile phosphorothioate (sVw, theoretical mass of 6729.099 Da). Its percentage is 0.21%.

Impurity 6 has a molecular mass of 6028.889 Da, formally corresponding to the deletion of 2′‐methoxyethylribose‐guanine (Gw, theoretical mass of 6028.877 Da) from Impurity 1. The retention time is 8.01 min, and the percent abundance is 0.16%.

Impurity 7, having a mass of 5932.940 Da, derives from the deletion of the tri‐nucleotide at the 3′ end, that is, sGw‐pMw‐sVw (theoretical mass of 5932.934 Da). Its retention time is 8.16 min, and the relative abundance is 0.18%.

Impurity 8 has a mass of 5625.813 Da. This corresponds to the deletion of the four 3′‐terminal residues, namely, an additional methoxyethylribose‐adenine‐methoxyethylribose‐guanine phosphorothioate (Aw‐sGw) besides the dinucleotide pMw‐sVw missing in Impurity 1 (theoretical mass: 5625.806 Da). The retention time of Impurity 8 is 7.90 min, whereas the calculated abundance is 0.22%.

Impurity 9, having a molecular mass of 5545.847 Da, is strictly related to Impurity 8, from which it differs by phosphate residue (−79.966 Da). In this case, the fourth residue from the 3′ end is missing as a nucleotide instead of a nucleoside as in Impurity 8. The theoretical mass is 5545.839 Da, the retention time 8.05 min, and the percent abundance is 0.19%.

Impurity 10, with an exact mass of 5152.771 Da, is indicative of the further deletion of the fifth nucleotide from the 3′ end, that is, methoxyethylribose‐methylcytosine phosphorothioate (sMw). The theoretical mass is 5152.763 Da, the retention time 7.97 min, and the abundance is 0.22%.

Impurity 11 has a molecular mass of 4919.678 Da and differs from Impurity 10 by −233.093 Da, corresponding to the additional deletion of the adjacent nucleoside, deoxyribosyl adenine (dA; theoretical mass of 4919.672). This is the most abundant impurity in the sample, with a percentage amount of 1.02%. The retention time is 7.78 min.

Impurity 12 has an observed mass of 4903.703 Da and differs from Impurity 11 by −15.976 Da, which is consistent with the conversion of phosphorothioate to phosphate. Its measured abundance is 0.10%, and its retention time is 7.76 min.

Impurity 13 remains unidentified; its molecular mass is 4679.715 Da, its retention time is 7.84 min, and its abundance is 0.55%.

Impurity 14 has a mass of 4599.657 Da, which formally corresponds to the deletion of a further nucleotide, 2′‐deoxythymine phosphorothioate, from the 3′ end of Impurity 11 (theoretical mass of 4599.649 Da). It has a retention time of 7.72 min, and its relative abundance was found to be 0.14%.

Impurity 15 is another unidentified impurity, having a molecular mass of 4564.653 Da, a relative abundance of 0.59% and a retention time of 7.81 min.

## 4 Conclusions

The QC of therapeutic ASOs poses specific challenges related to the high number of product‐related impurities with a high degree of similarity in structure, which may arise from both SPS and degradation pathways. These impurities are difficult to separate by chromatography and often present coelution issues. The objective of this work was to optimise an IP‐RP‐UHPLC‐HRMS method with ESI(−) equipped with Orbitrap technology for the characterisation of the ASOs fomivirsen and tofersen. The main experimental issue was to find the optimal balance between chromatographic resolution and MS sensitivity. This was achieved by using DoE as a fundamental aid for investigating the relevant factors on several chromatographic responses related to sensitivity (peak heights), efficiency (peak widths) and selectivity (resolution between selected peak pairs), highlighting their interactions and quadratic effects.

The ATP was approached by a rational and systematic methodology. First, the objective of the knowledge management step was to determine the most suitable alkylamine to be utilised as an ion coupling agent and to establish fundamental MS parameters settings. A combination of DIPEA and HFIP was used as an ion‐pairing system, and shared MS parameters were selected for both FMV and TFR analyses. These were based on MS signal intensity of the API and on the quality of MS spectrum profile. Subsequently, a comprehensive investigation was conducted utilising DoE to optimise the concentration of DIPEA, the concentration of HFIP and the slope of the mobile phase flow gradient. RSM, through the use of Box–Behnken design and multicriteria decision tools, was found to be an effective approach in leading the study towards identifying optimal conditions for FMV and TFR analysis. The analysis of contour plots facilitated the delineation of a multidimensional space, the sweet spot, in which the requisites for the responses are satisfied, thereby enabling their simultaneous optimisation. The present work is in line with the recent trends supporting a structured framework in the development of pharmaceutical analytical procedures, by applying an enhanced multivariate approach in the field of therapeutic ASO characterisation. This approach was implemented in the characterisation of four and thirteen impurities of FMV and TFR, respectively.

## Funding

The authors have nothing to report.

## Supporting information


**Table S1:** Optimised chromatographic and mass acquisition parameters.
**Table S2:** Experiments run in the scouting phase.
**Table S3:** ANOVA for RSM models.
**Table S4:** Quality parameters of the models.
**Table S5:** Responses: RSM statistics, target values and measured and predicted responses under the optimised conditions.
**Figure S1:** Scouting experiments: Graphical visualisation of MS peak heights for (a) fomivirsen and (b) tofersen. Experiments E1–E6 (blue bars): 5 mM TEA; Experiments E7–E13 (orange bars): 5 mM DBA; Experiments E14–E15: 4 mM DIPEA (green bars). Full details of experimental conditions are explained in Table S2.
**Figure S2:** Total ion chromatograms and ESI negative mass spectra of fomivirsen using (a) TEA Experiment E4, (b) DBA Experiment E12 and (c) DIPEA Experiment E15. Full details of experimental conditions are explained in Table S2.
**Figure S3:** Total ion chromatograms and ESI negative mass spectra of tofersen using (a) TEA Experiment E4, (b) DBA Experiment E12 and (c) DIPEA Experiment E15. Full details of experimental conditions are explained in Table S2.
**Figure S4:** Box–Behnken design observed *versus* predicted plots. (a) FMV peak height (h_F_), (b) FMV baseline peak width (w_F_) and (c) R_12F_ (Resolution FMV Impurity 1/FMV Impurity 2).
**Figure S5:** Box–Behnken design graphical analysis of effects. (a) FMV peak height (h_F_), (b) FMV baseline peak width (w_F_) and (c) R_12F_ (Resolution FMV Impurity 1/FMV Impurity 2).
**Figure S6:** Box–Behnken design observed *versus* predicted plots. (a) TFR peak height (h_T_), (b) TFR baseline peak width (w_T_), (c) R_12T_ (resolution TFR Impurity 1/TFR Impurity 2) and (d) R_23T_ (resolution TFR Impurity 2/TFR Impurity 3).
**Figure S7:** Box–Behnken graphical analysis of effects. (a) TFR peak height (h_T_), (b) TFR baseline peak width (w_T_), (c) R_12T_ (resolution TFR Impurity 1/TFR Impurity 2) and (d) R_23T_ (resolution TFR Impurity 2/TRF Impurity 3).

## Data Availability

The data that support the findings of this study are available from the corresponding author upon reasonable request.
