# Supplementary material for: Characterisation of Antisense Oligonucleotides by Ion‐Pair Reversed‐Phase UHPLC‐HRMS: Method development using Design of Experiments
Source: J Mass Spectrom. 2026 Mar 19;61(4):e70049. doi: 10.1002/jms.70049 (PMC13002315; doi:10.1002/jms.70049)

**SUPPORTING INFORMATION**

**Characterization of Antisense Oligonucleotides by Ion-Pair Reversed-Phase UHPLC-HRMS: Method development using Design of Experiments**

Antonio Triolo^1^, Fabiana Tavani^1^, Prisca Barnini^2^, Sandra Furlanetto^2^, Serena Orlandini^2,^*

^1^Laboratory of Advanced Analytics, Menarini Ricerche Spa, Via Rismondo 12A, Florence, Italy ^2^Department of Chemistry “Ugo Schiff”, University of Florence, Via U. Schiff 6, Sesto Fiorentino, Florence, Italy

**SUPPLEMENTARY TABLES:**

**TABLE S1** | Optimized chromatographic and mass acquisition parameters.

**TABLE S2** | Experiments run in the scouting phase.

**TABLE S3** | ANOVA for RSM models.

**TABLE S4** | Quality parameters of the models.

**TABLE S5** | Responses: RSM statistics, target values, measured and predicted responses under the optimised conditions.

**SUPPLEMENTARY FIGURES:**

**FIGURE S1** | Scouting experiments: graphical visualization of MS peak heights for (a) Fomivirsen and (b) Tofersen.

**FIGURE S2** | Total Ion Chromatograms and ESI negative mass spectra of Fomivirsen using (a) TEA Experiment E4, (b) DBA Experiment E12, (c) DIPEA Experiment E15 (see Table S2 for detailed experimental conditions).

**FIGURE S3** | Total Ion Chromatograms and ESI negative mass spectra of Tofersen using (a) TEA Experiment E4, (b) DBA Experiment E12, (c) DIPEA Experiment E15 (see Table S2 for detailed experimental conditions).

**FIGURE S4** | Box-Behnken design observed *versus* predicted plots for Fomivirsen

**FIGURE S5** | Box-Behnken design graphical analysis of effects for Fomivirsen.

**FIGURE S6** | Box-Behnken design observed *versus* predicted plots for Tofersen.

**FIGURE S7** | Box-Behnken graphical analysis of effects for Tofersen.

**TABLE S1** | Optimized chromatographic and mass acquisition parameters.

| **Parameters** | **Settings** |
| --- | --- |
| *Chromatography* |  |
| System | Vanquish Horizon UHPLC |
| Column | Biozen Oligo column (2.1 x 100 mm, 1.7 µm)  core-shell particle bonded with a C18 stationary phase |
| Column temperature | 40 °C |
| Mobile Phase | FMV: (A) 4 mM DIPEA, 58 mM HFIP in ultrapure water;  (B) 4 mM DIPEA, 58 mM HFIP in methanol  TFR: (A) 7 mM DIPEA, 52 mM HFIP in ultrapure water;  (B) 7 mM DIPEA, 52 mM HFIP in methanol |
| Injection volume | 2 μL |
| Flow rate | 0.20 mL min^-1^ |
| Run time | 21.5 min |
| Gradient | 5-80% (B)  FMV: gradient slope 3.7 %B/min  TFR: gradient slope 4.5% B/min |
| *Mass spectrometry* |  |
| System | Q-Exactive Plus BioPharma |
| Ionization mode | ESI (-) |
| Capillary voltage | -2800 V |
| Capillary temperature | 300 °C |
| Sheath gas | 60 units |
| Auxiliary gas | 20 units |
| Auxiliary gas temperature | 400 °C |
| S-lens | 90 V |
| Source CID | 0 V |
| Normalized collision energy (NCE) | 13-15-17 V |
| Acquisition range MS scan *m/z* | 550-2500 |
| Orbitrap Full MS resolution | 70000 |
| MS AGC target | 1·10^6^ |

HFIP, 1,1,1,3,3,3-hexafluoroisopropanol; DIPEA, N,N-diisopropylethylamine

**TABLE S2** | Experiments run in the scouting phase.

|  | | | | | | **Fomivirsen** | | **Tofersen** | |
| --- | --- | --- | --- | --- | --- | --- | --- | --- | --- |
| **Exp. no.** | **Alkylamine type and concentration** | **Sheath gas (units)** | **Auxiliary gas temperature (°C)** | **S-lens (V)** | **Source CID (V)** | **MS peak height (counts)** | **MS spectrum quality** | **MS peak height (counts)** | **MS spectrum quality** |
| E1 | 5 mM TEA | 40 | 320 | 60 | 0 | 1.10E+08 | Good | 7.78E+08 | Good |
| E2 | 5 mM TEA | 60 | 320 | 60 | 0 | 3.61E+08 | Good | 6.91E+08 | Good |
| E3 | 5 mM TEA | 60 | 400 | 60 | 0 | 1.75E+09 | Good | 2.46E+09 | Good |
| E4 | 5 mM TEA | 60 | 400 | 90 | 0 | 2.20E+09 | Good | 2.33E+09 | Good |
| E5 | 5 mM TEA | 60 | 450 | 90 | 0 | 1.56E+09 | Good | 1.98E+09 | Good |
| E6 | 5 mM TEA | 60 | 500 | 90 | 0 | 1.72E+09 | Good | 2.08E+09 | Poor |
| E7 | 5 mM DBA | 60 | 400 | 90 | 0 | 1.11E+09 | Poor | 1.00E+09 | Poor |
| E8 | 5 mM DBA | 60 | 400 | 90 | 10 | 1.11E+09 | Poor | 8.66E+08 | Poor |
| E9 | 5 mM DBA | 60 | 400 | 90 | 5 | 1.03E+09 | Poor | 1.22E+09 | Poor |
| E10 | 5 mM DBA | 60 | 450 | 90 | 0 | 1.01E+09 | Poor | 8.74E+08 | Poor |
| E11 | 5 mM DBA | 60 | 500 | 90 | 0 | 9.61E+08 | Poor | 8.62E+08 | Poor |
| E12 | 5 mM DBA | 60 | 450 | 90 | 5 | 1.12E+09 | Poor | 1.08E+09 | Poor |
| E13 | 5 mM DBA | 60 | 500 | 90 | 5 | 1.17E+09 | Poor | 9.93E+08 | Poor |
| E14 | 4 mM DIPEA | 60 | 400 | 90 | 5 | 4.95E+09 | Good | 4.06E+09 | Good |
| E15 | 4 mM DIPEA | 60 | 400 | 90 | 0 | 4.40E+09 | Good | 4.09E+09 | Good |

Constant experimental parameters: HFIP concentration, 12.5 mM; column temperature, 40° C; flow rate, 0.20 mL min^-1^; gradient, 5-80 %B in 21.5 min; high voltage, -2800 V; auxiliary gas, 20 units; capillary temperature, 300 °C, full MS resolution, 70000; MS AGC Target, 1·10^6^; MS scan *m/z*, 550-2500.

**TABLE S3** | ANOVA for RSM models.

| **Source of variation** | **Sum of Squares** | **Degrees of freedom** | **Mean square** | **F ratio** | **p value** |
| --- | --- | --- | --- | --- | --- |
| **h_F_** |  |  |  |  |  |
| Regression | 2.012·10^4^ | 5 | 4.024·10^3^ | 1.78·10 | 0.000 |
| Residual | 2.035·10^3^ | 9 | 2.261·10^2^ |  |  |
| Lack of Fit | 5.879·10^2^ | 7 | 8.398·10 | 1.16·10^-1^ | 0.987 |
| Pure error | 1.447·10^3^ | 2 | 7.234·10^2^ |  |  |
| **w_F_** |  |  |  |  |  |
| Regression | 7.006·10^-3^ | 9 | 7.785·10^-4^ | 7.50·10 | 0.000 |
| Residual | 5.192·10^-5^ | 5 | 1.038·10^-5^ |  |  |
| Lack of Fit | 3.250·10^-6^ | 3 | 1.083·10^-6^ | 4.45·10^-2^ | 0.984 |
| Pure error | 4.867·10^-5^ | 2 | 2.433·10^-5^ |  |  |
| **R_12F_** |  |  |  |  |  |
| Regression | 3.202·10^-1^ | 5 | 6.405·10^-2^ | 1.02·10 | 0.002 |
| Residual | 5.634·10^-2^ | 9 | 6.260·10^-3^ |  |  |
| Lack of Fit | 3.998·10^-2^ | 7 | 5.711·10^-3^ | 6.98·10^-1^ | 0.699 |
| Pure error | 1.636·10^-2^ | 2 | 8.182·10^-3^ |  |  |
| **h_T_** |  |  |  |  |  |
| Regression | 3.247·10^3^ | 7 | 4.639·10^2^ | 1.29·10 | 0.002 |
| Residual | 2.526·10^2^ | 7 | 3.608·10 |  |  |
| Lack of Fit | 6.979·10 | 5 | 1.396·10 | 1.53·10^-1^ | 0.960 |
| Pure error | 1.828·10^2^ | 2 | 9.140·10 |  |  |
| **w_T_** |  |  |  |  |  |
| Regression | 2.610·10^-3^ | 6 | 4.350·10^-4^ | 4.32·10 | 0.000 |
| Residual | 8.061·10^-5^ | 8 | 1.008·10^-5^ |  |  |
| Lack of Fit | 7.944·10^-5^ | 6 | 1.324·10^-5^ | 2.27·10 | 0.043 |
| Pure error | 1.167·10^-6^ | 2 | 5.833·10^-7^ |  |  |
| **R_12T_** |  |  |  |  |  |
| Regression | 3.20512·10^-1^ | 6 | 5.34186·10^-2^ | 2.62·10 | 0.000 |
| Residual | 1.63161·10^-2^ | 8 | 2.03951·10^-3^ |  |  |
| Lack of Fit | 1.13754·10^-2^ | 6 | 1.89590·10^-3^ | 7.67·10^-1^ | 0.661 |
| Pure error | 4.94067·10^-3^ | 2 | 2.47033·10^-3^ |  |  |
| **R_23T_** |  |  |  |  |  |
| Regression | 2.84434·10^-1^ | 6 | 4.74057·10^-2^ | 1.47·10 | 0.001 |
| Residual | 2.58519·10^-2^ | 8 | 3.23149·10^-3^ |  |  |
| Lack of Fit | 2.36413·10^-2^ | 6 | 3.94021·10^-3^ | 3.56 | 0.235 |
| Pure error | 2.21067·10^-3^ | 2 | 1.10533·10^-3^ |  |  |

**TABLE S4** | Quality parameters of the models.

|  | **Fomivirsen** | | | **Tofersen** | | | |
| --- | --- | --- | --- | --- | --- | --- | --- |
|  | **h_F_** | **w_F_** | **R_12F_** | **h_T_** | **w_T_** | **R_12T_** | **R_23T_** |
| R^2^ | 0.9082 | 0.9926 | 0.8504 | 0.9278 | 0.9700 | 0.9516 | 0.9167 |
| R^2^_adj_ | 0.8571 | 0.9794 | 0.7673 | 0.8557 | 0.9476 | 0.9152 | 0.8542 |
| Q^2^ | 0.8121 | 0.9771 | 0.5299 | 0.7881 | 0.8394 | 0.8143 | 0.5866 |
| Reproducibility^a^ | 0.5428 | 0.9517 | 0.6958 | 0.6344 | 0.9970 | 0.8973 | 0.9501 |

Abbreviations: h_F_, FMV peak height; w_F_, FMV baseline peak width; R_12F_, resolution FMV Impurity 1/FMV Impurity 2; h_T_, TFR peak height; w_T_, TFR baseline peak width; R_12T_, resolution TFR Impurity 1/TFR Impurity 2; R_23T_, resolution TFR Impurity 2/TFR Impurity 3. ^a^Reproducibility = 1 - MS_pe_/MS_tot_, where MS_pe_ is the mean square of the pure error and MS_tot_ the total mean square of the responses Y.

**TABLE S5** | Responses: RSM statistics, target values, measured and predicted responses under the optimised conditions.

|  | **Fomivirsen** | | | **Tofersen** | | | |
| --- | --- | --- | --- | --- | --- | --- | --- |
|  | **h_F_**  **(counts)** | **w_F_**  **(min)** | **R_12F_** | **h_T_**  **(counts)** | **w_T_**  **(min)** | **R_12T_** | **R_23T_** |
| RSM minimum | 173.48 | 0.082 | 0.048 | 104.10 | 0.080 | 0.589 | 0.100 |
| RSM maximum | 288.81 | 0.158 | 0.560 | 160.62 | 0.113 | 1.119 | 0.597 |
| RSM median | 248.00 | 0.104 | 0.178 | 135.77 | 0.093 | 0.813 | 0.295 |
| Target | 275.00 | 0.100 | 0.280 | 145.00 | 0.095 | 0.600 | 0.300 |
| Optimum^a^ measured value | 289.05 | 0.085 | 0.271 | 144.04 | 0.091 | 0.772 | 0.287 |
| Optimum predicted value | 280.10 | 0.083 | 0.286 | 148.44 | 0.080 | 0.716 | 0.350 |
| Optimum predicted intervals | 260.03-300.18 | 0.077-0.090 | 0.167-0.405 | 141.13-155.75 | 0.076-0.084 | 0.662-0.770 | 0.281-0.420 |

Abbreviations: h_F_, FMV peak height; w_F_, FMV baseline peak width; R_12F_, resolution FMV Impurity 1/FMV Impurity 2; h_T_, TFR peak height;

w_T_, TFR baseline peak width; R_12T_, resolution TFR Impurity 1/TFR Impurity 2; R_23T_, resolution TFR Impurity 2/TFR Impurity 3.

^a^Optimum (average data, *n*=2): for FMV, 4 mM DIPEA, 58 mM HFIP and 3.7 %B/min; for TFR, 7 mM DIPEA, 52 mM HFIP and 4.5 %B/min.

**FIGURE S1** | Scouting experiments: graphical visualization of MS peak heights for (a) Fomivirsen and (b) Tofersen. Exp. E1-E6 (blue bars): 5 mM TEA; Exp. E7-E13 (orange bars): 5 mM DBA; Exp. E14-E15: 4 mM DIPEA (green bars). Full details of experimental conditions are explained in Table S2.


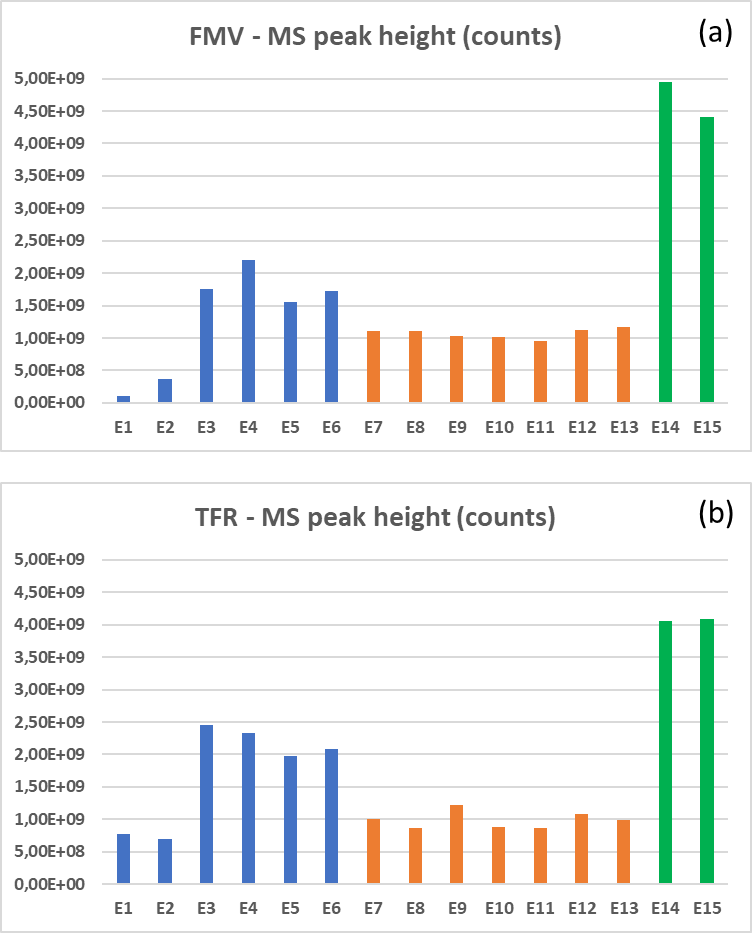


**FIGURE S2** | Total Ion Chromatograms and ESI negative mass spectra of Fomivirsen using (a) TEA Experiment E4, (b) DBA Experiment E12, (c) DIPEA Experiment E15. Full details of experimental conditions are explained in Table S2.


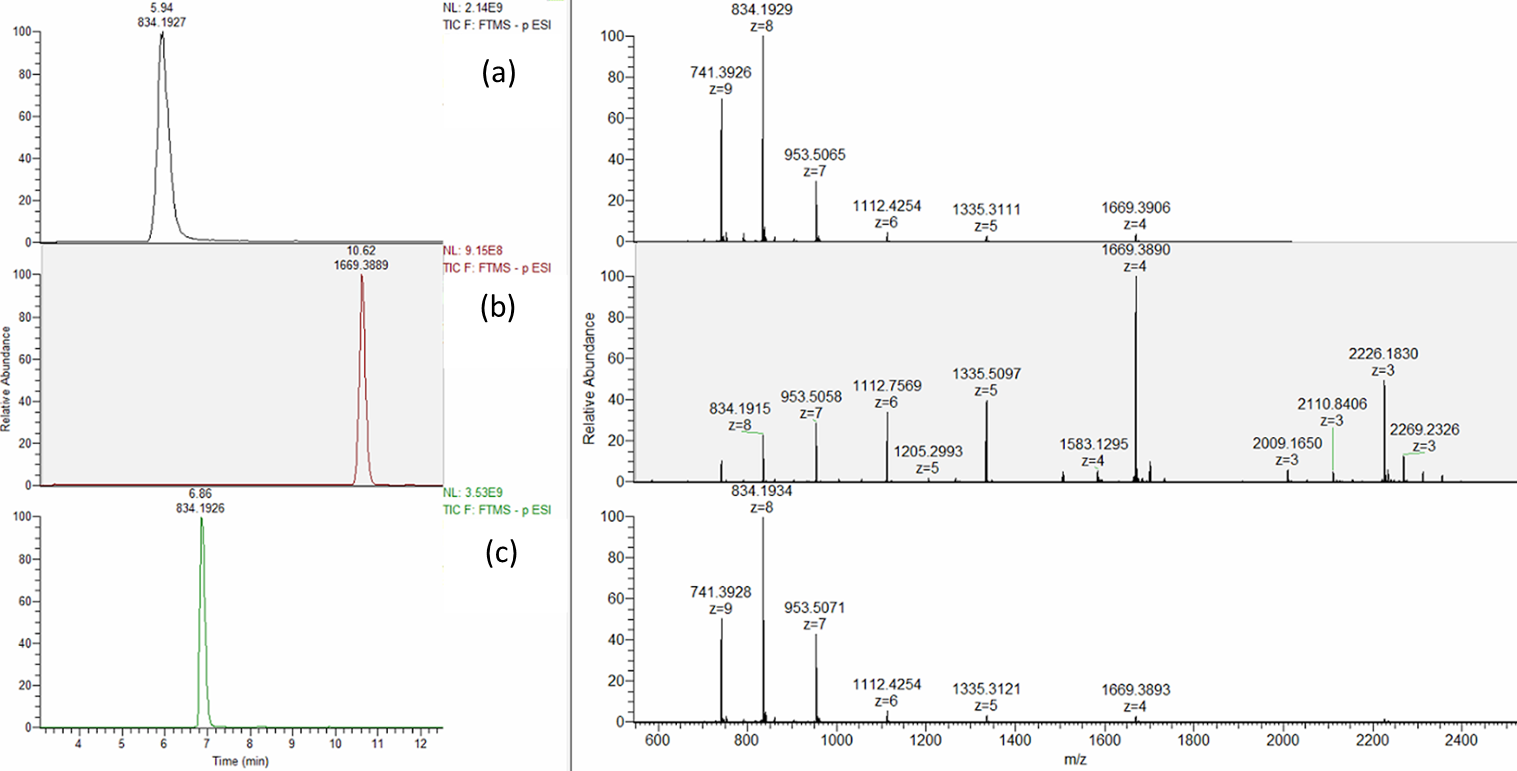


**FIGURE S3** | Total Ion Chromatograms and ESI negative mass spectra of Tofersen using (a) TEA Experiment E4, (b) DBA Experiment E12, (c) DIPEA Experiment E15. Full details of experimental conditions are explained in Table S2.


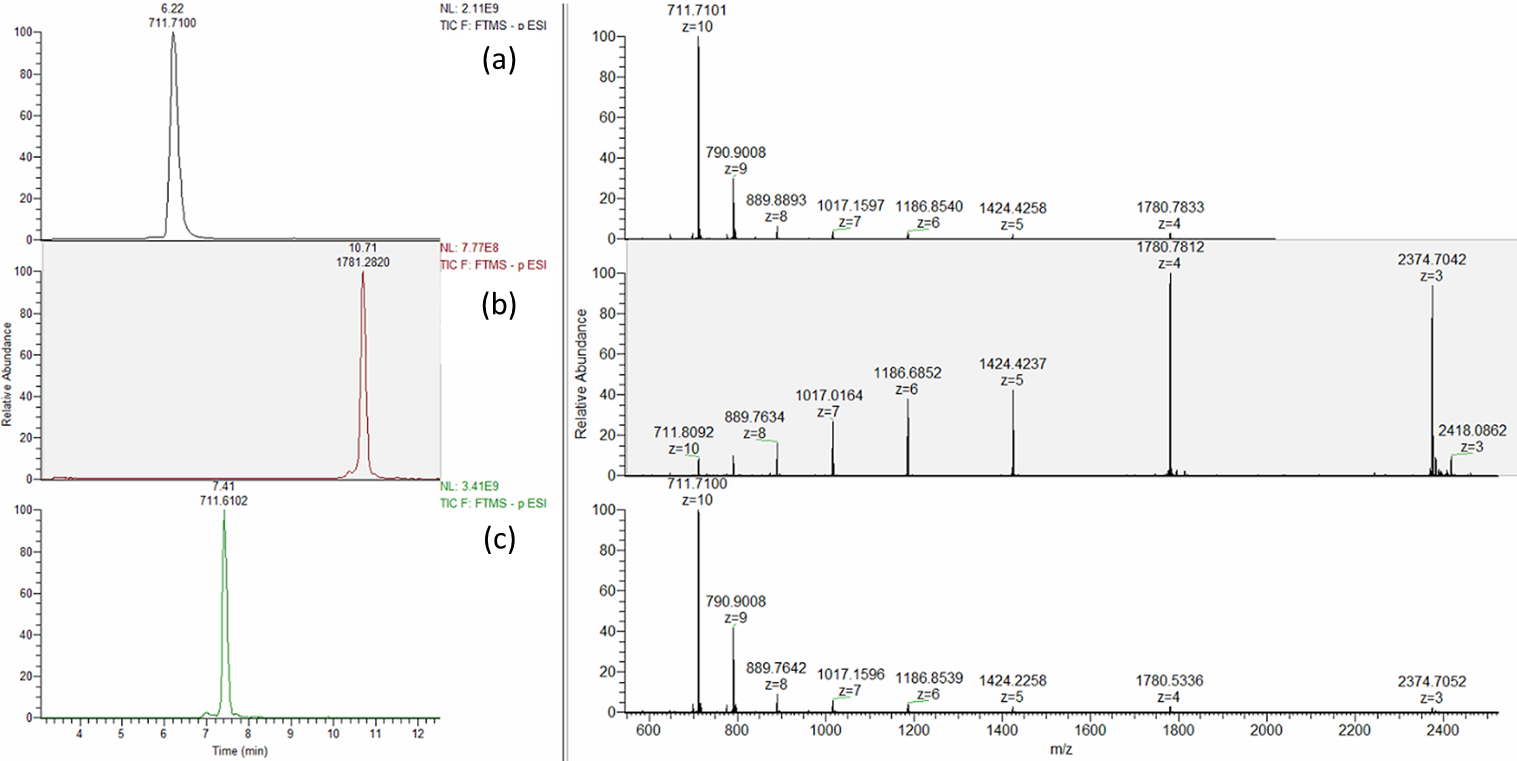


**FIGURE S4** | Box-Behnken observed *vs.* predicted plots. (a) FMV peak height (h_F_), (b) FMV baseline peak width (w_F_), (c) R_12F_ (Resolution FMV Impurity 1/FMV Impurity 2).

**
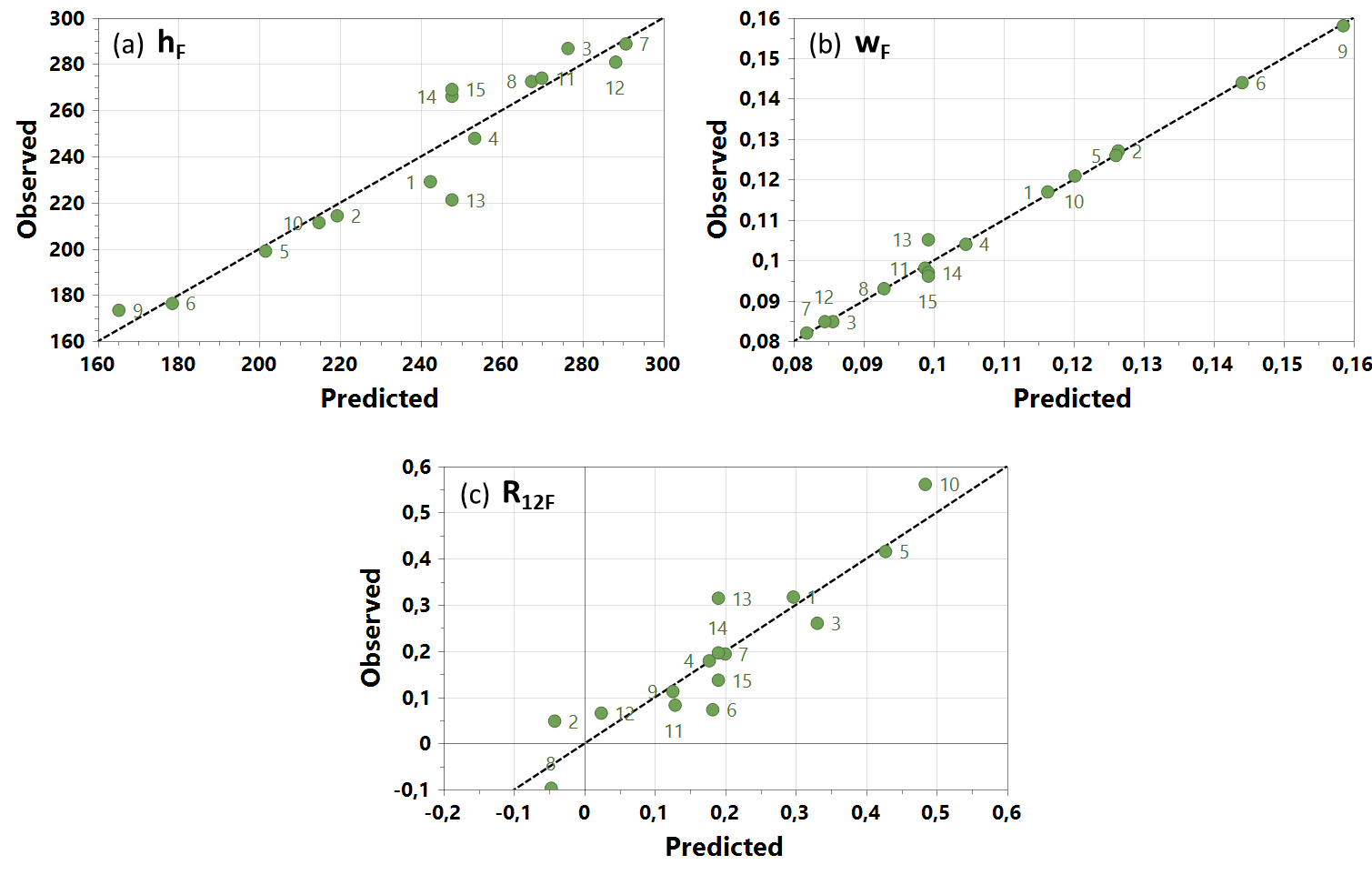
**

**FIGURE S5** | Box-Behnken graphical analysis of effects. (a) FMV peak height (h_F_), (b) FMV baseline peak width (w_F_), (c) R_12F_ (Resolution FMV Impurity 1/FMV Impurity 2).


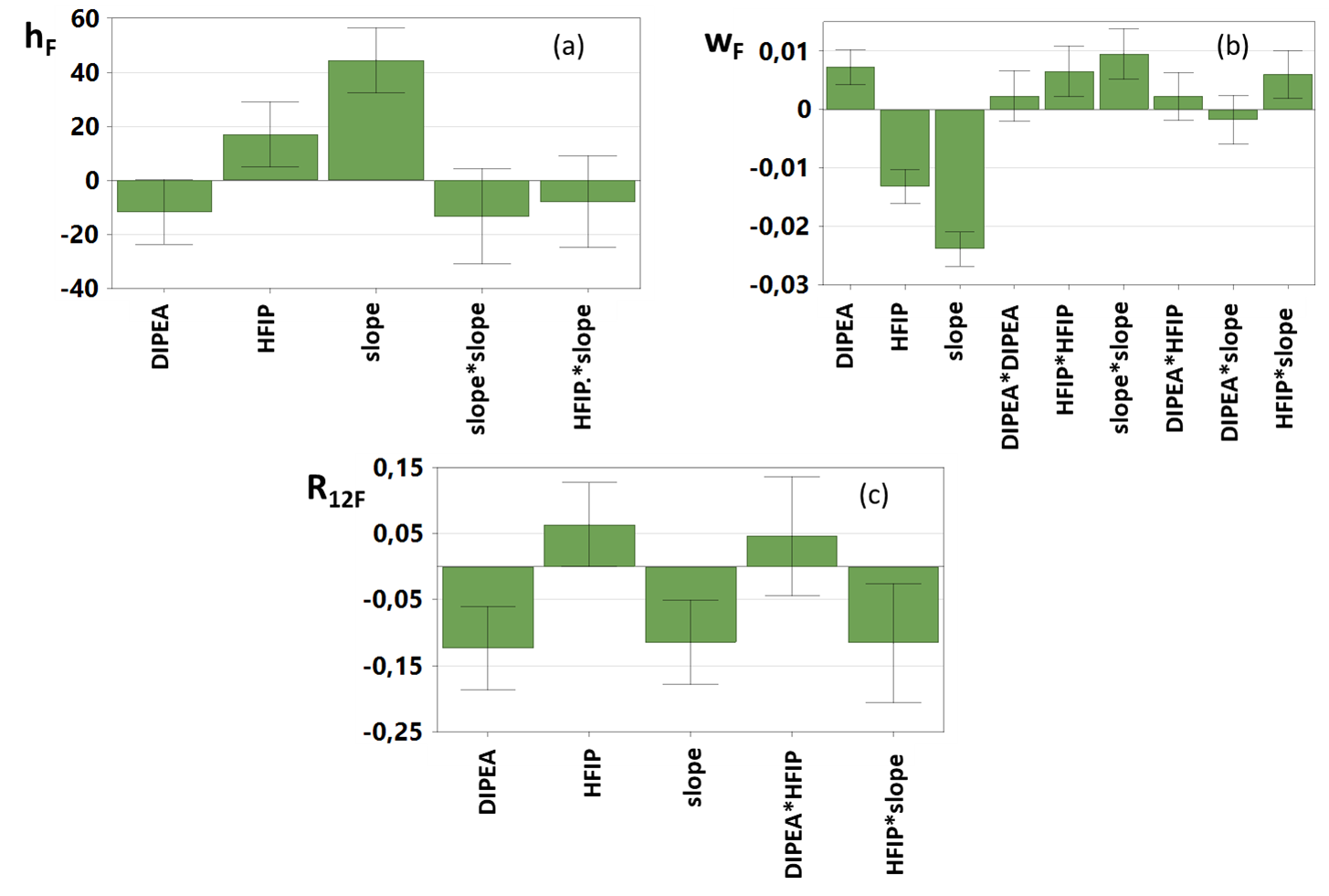


**FIGURE S6** | Box-Behnken observed *vs.* predicted plots. (a) TFR peak height (h_T_), (b) TFR baseline peak width (w_T_), (c) R_12T_ (resolution TFR Impurity 1/TFR Impurity 2), (d) R_23T_ (resolution TFR Impurity 2/TFR Impurity 3).

**
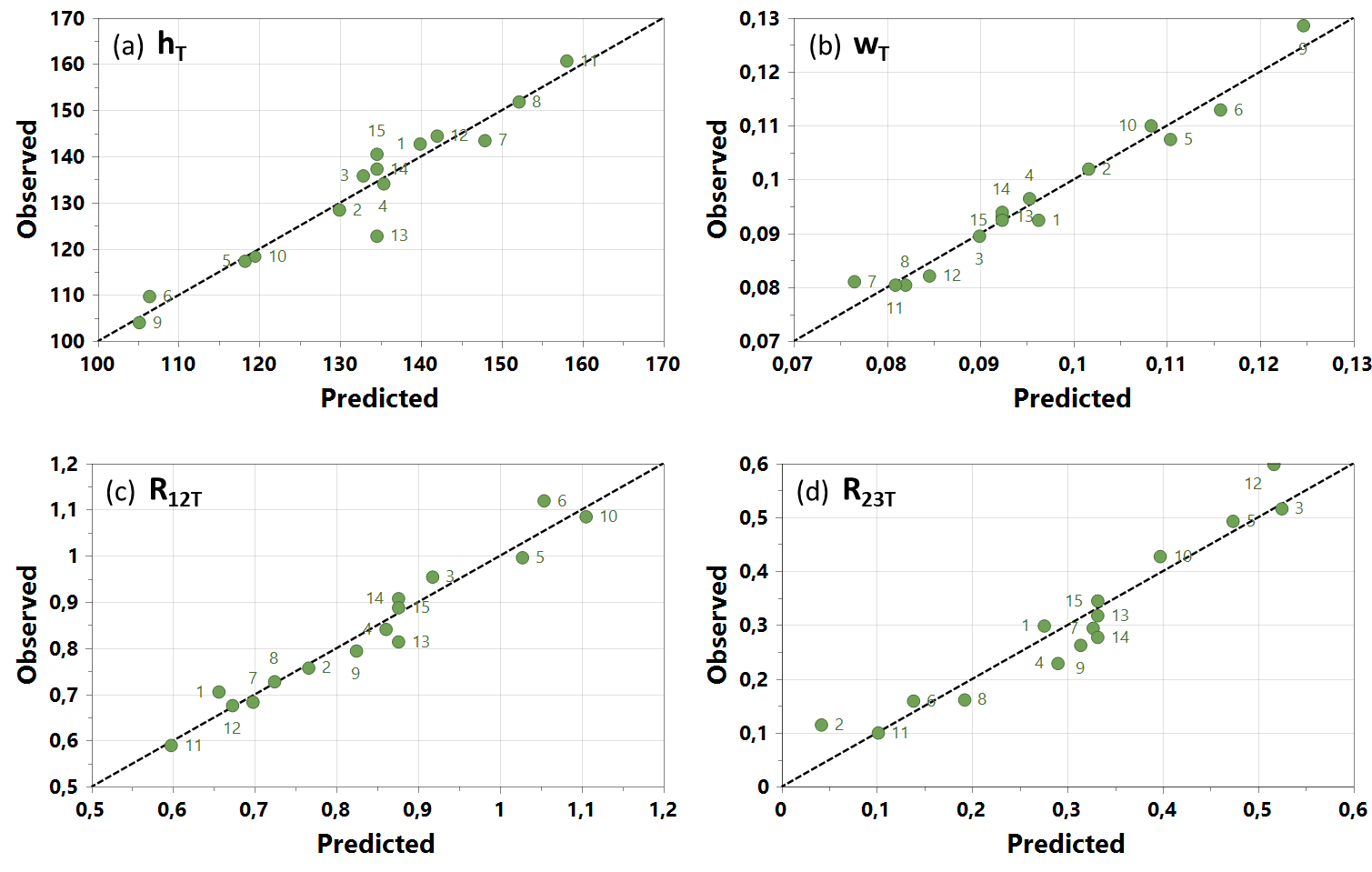
**

**FIGURE S7** | Box-Behnken graphical analysis of effects. (a) TFR peak height (h_T_), (b) TFR baseline peak width (w_T_), (c) R_12T_ (resolution TFR Impurity 1/TFR Impurity 2), (d) R_23T_ (resolution TFR Impurity 2/TRF Impurity 3).


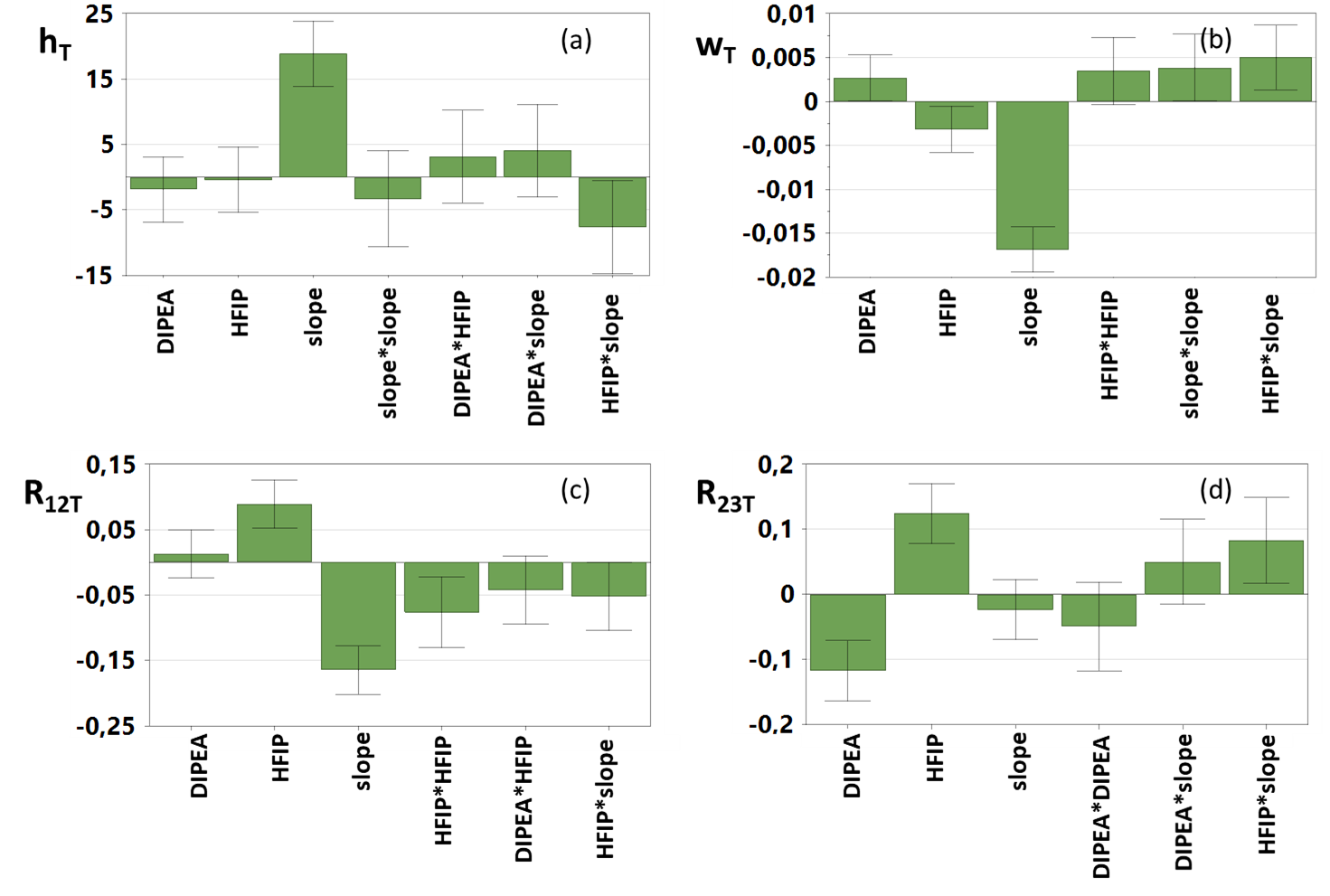

Supplement: Supplementary file 1 — Table S1: Optimised chromatographic and mass acquisition parameters. Table S2: Experiments run in the scouting phase. Table S3: ANOVA for RSM models. Table S4: Quality parameters of the models. Table S5: Responses: RSM statistics, target values and measured and predicted responses under the optimised conditions. Figure S1: Scouting experiments: Graphical visualisation of MS peak heights for (a) fomivirsen and (b) tofersen. Experiments E1–E6 (blue bars): 5 mM TEA; Experiments E7–E13 (orange bars): 5 mM DBA; Experiments E14–E15: 4 mM DIPEA (green bars). Full details of experimental conditions are explained in Table S2. Figure S2: Total ion chromatograms and ESI negative mass spectra of fomivirsen using (a) TEA Experiment E4, (b) DBA Experiment E12 and (c) DIPEA Experiment E15. Full details of experimental conditions are explained in Table S2. Figure S3: Total ion chromatograms and ESI negative mass spectra of tofersen using (a) TEA Experiment E4, (b) DBA Experiment E12 and (c) DIPEA Experiment E15. Full details of experimental conditions are explained in Table S2. Figure S4: Box–Behnken design observed versus predicted plots. (a) FMV peak height (hF), (b) FMV baseline peak width (wF) and (c) R12F (Resolution FMV Impurity 1/FMV Impurity 2). Figure S5: Box–Behnken design graphical analysis of effects. (a) FMV peak height (hF), (b) FMV baseline peak width (wF) and (c) R12F (Resolution FMV Impurity 1/FMV Impurity 2). Figure S6: Box–Behnken design observed versus predicted plots. (a) TFR peak height (hT), (b) TFR baseline peak width (wT), (c) R12T (resolution TFR Impurity 1/TFR Impurity 2) and (d) R23T (resolution TFR Impurity 2/TFR Impurity 3). Figure S7: Box–Behnken graphical analysis of effects. (a) TFR peak height (hT), (b) TFR baseline peak width (wT), (c) R12T (resolution TFR Impurity 1/TFR Impurity 2) and (d) R23T (resolution TFR Impurity 2/TRF Impurity 3). [file JMS-61-e70049-s001.docx]
